# Targeting gut microbiota dysbiosis in inflammatory bowel disease: a systematic review of current evidence

**DOI:** 10.3389/fmed.2025.1435030

**Published:** 2025-02-18

**Authors:** Asmaa Farah, Pradipta Paul, Anfal Sher Khan, Aparajita Sarkar, Sa’ad Laws, Ali Chaari

**Affiliations:** Weill Cornell Medicine–Qatar, Qatar Foundation, Education City, Doha, Qatar

**Keywords:** Crohn’s disease, ulcerative colitis, inflammation, microbiology, gastroenterology

## Abstract

**Introduction:**

The dysbiosis of the gut microbiota has been identified as a central factor in the pathogenesis of inflammatory bowel disease (IBD), a chronic condition characterized by frequent recurrence and various adverse effects of traditional therapies. While treatments targeting the gut microbiota show promise, their efficacy in IBD management still requires extensive evaluation. Our systematic review analyzes recent studies to elucidate the advancements and challenges in treating IBD using microbial-based therapies.

**Methods:**

Through a comprehensive systematic review spanning key scientific databases—PubMed, Embase, Cochrane, Web of Science, Scopus, and Google Scholar—we scrutinized the impact of probiotics, prebiotics, synbiotics, and fecal microbiota transplantation (FMT) on individuals with IBD. Our detailed analysis covered study and participant demographics, along with seven key outcome measures: disease activity index, inflammatory markers, serum cytokines, microbiome composition, adverse effects, and the rates of remission and relapse.

**Results:**

From 6,080 initial search hits, we included 71 studies that assessed various interventions compared to placebo or standard medical therapy. Although there was notable variation in clinical results while assessing different outcomes, overall, probiotics, prebiotics, and synbiotics enhanced the success rates in inducing remission among IBD patients. Furthermore, we noted significant reductions in levels of pro-inflammatory markers and cytokines. Additionally, the requirement for steroids, hospitalization, and poor outcomes in endoscopic and histological scores were significantly reduced in individuals undergoing FMT.

**Conclusion:**

Our investigation highlights the potential of targeting gut microbiota dysbiosis with microbial-based therapies in patients with IBD. We recommend conducting larger, placebo-controlled randomized trials with extended follow-up periods to thoroughly assess these treatments’ clinical efficacy and safety before widespread recommendations for clinical application.

## 1 Introduction

Inflammatory bowel disease (IBD), which includes Crohn’s disease (CD) and ulcerative colitis (UC), is a chronic condition characterized by recurring inflammation of the gastrointestinal tract (1). Data from 2017 estimate that 6.8 million individuals worldwide suffer from IBD, with an age-standardized prevalence rate of 84.3 per 100,000 individuals, revealing an upward trend from the previous census (2). The growing prevalence of IBD, particularly notable in industrialized countries, signals a looming socioeconomic challenge (3). Although the root causes of IBD remain elusive, the research underscores the significant roles of genetic factors, environmental conditions (4) and the gut microbiome—a vital community of microorganisms integral to host health and disease development (5). Dysbiosis, or the disruption of the gut microbiome, has been strongly linked to the development of IBD, propelling interest in therapeutic strategies to rectify this imbalance (6). Despite the potential of these treatments, the effectiveness of interventions targeting dysbiosis in IBD patients remains subject to thorough investigation. This underscores the urgent need to examine the impacts of these interventions on dysbiosis to gain a deeper understanding of both the progress and challenges in this area of research.

The gut microbiome comprises more than a thousand species of bacteria, viruses, and fungi, forming a community that can influence the physiology of host health and disease (5). The symbiotic relationships among these microbial species contribute to vital functions, including digestion, immune system modulation, and protection against pathogenic invaders (5). However, disruptions to this finely balanced microbiome can lead to significant imbalances in the composition of the microbiota. Such disturbances result in changes in the distribution of microbial species, which in turn can profoundly affect the host’s health (7).

The gut microbiota was found to be regulated by the melanocortin system through the gut-brain-adrenal axis. The melanocortin system, which involves melanocortin receptors and their ligands such as adrenocorticotropic hormone (ACTH) and melanocyte-stimulating hormones (MSHs), plays a significant role in this system (8). The mechanism involves activation of the Hypothalamic–Pituitary–Adrenal (HPA) Axis. The activation of this system can lead to the release of cortisol from the adrenal glands. Cortisol, a glucocorticoid, can influence the gut microbiota composition by altering gut permeability and immune function, thereby affecting the balance of microbial species in the gut. Furthermore, chronic stress or activation of the HPA axis can lead to dysbiosis, an imbalance in the gut microbiota that is often associated with various gastrointestinal disorders (9). The melanocortin system also has anti-inflammatory effects, mediated by melanocortin receptors (particularly MC1R and MC3R). These receptors are expressed in various tissues, including immune cells, where they modulate inflammatory responses. The direct role of melanocortin in the regulation of inflammatory processes has emerged from their potential to inhibit the family of NF-κB involved in the transcriptional regulation of many genes involved in the synthesis of cytokines (especially TNF) and related receptors (10, 11).

In addition, the interplay between the gut-brain axis in obesity may influence the development of IBD. Interestingly, the evidence suggests that the diversity of gut microbiota is significantly decreased in obese groups with an increased ratio of Firmicutes/Bacteroidetes. Furthermore, obesity-mediated gut microbiota alteration promotes lipopolysaccharide (LPS) producing bacteria, which in turn down regulates tight junctions and increases the permeability of the gut barrier and translocation of LPS. In this mechanism, LPS promote the release of proinflammatory cytokines leading to a state of low inflammation (10). This is often the cited association between obesity-mediated gut microbiota alterations and the increased risk of IBD.

Reflecting the complexity of the gut ecosystem, extensive research efforts have been directed toward understanding how specific groups within the microbiome contribute to the inflammatory processes underlying IBD (12). In a pivotal study, Sellon et al. demonstrated that resident enteric bacteria are essential for developing spontaneous colitis and immune system activation in IL-10-deficient mice, underscoring the gut microbiome’s pivotal role in inflammatory processes (13). Building on these findings, Lloyd-Price et al. adopted a multi-omics methodology to provide an integrative view of the complex interactions between the host and microbial communities in IBD across a year-long investigation. Their research reported a shift toward facultative anaerobes and changes in microbial gene expression, metabolite concentrations, and serum immunoglobulin levels, suggesting that such imbalances might influence IBD’s pathology (14). Additionally, Abdel-Rahman et al. identified a decrease in alpha diversity in CD and UC and a significant correlation between the *Fusobacterium* genus and CD, underlining the intricate link between microbial diversity and IBD pathogenesis (15). Collectively, these insights highlight the critical role of dysbiosis in IBD and propose that interventions aimed at modulating the gut microbiome might offer a promising avenue for treatment.

Exploring microbial agents as a therapeutic avenue in IBD has undergone extensive investigation through various clinical and experimental studies involving human and animal subjects (16). Among the promising treatment strategies, interventions with probiotics, prebiotics, synbiotics, and fecal microbiota transplantation (FMT) have gained increasing attention for their potential benefits. Probiotics are live beneficial microorganisms that contribute to gut health when administered in adequate amounts (17), and have been recognized for their safety and tolerability (18). Prebiotics consist of substrates that encourage the proliferation of beneficial intestinal microbes, amplifying their advantageous actions (19). Symbiotic represent a mixture of probiotics and prebiotics, incorporating live beneficial microorganisms and substrates that are selectively utilized by these host microbes (20). FMT refers to the transplantation of fecal matter from a healthy donor to the patient (21). Another highly discussed intervention, that is not the focus of the systematic review; however, plays a crucial role in the study of IBD therapeutics is diet. It has a profound impact on the composition and function of the gut microbiota, influencing overall health and susceptibility to various diseases. Research has shown that the Western diet and certain dietary components can disrupt the gut microbiota, damage the intestinal mucosal layer, and impair mucosal immunity, all of which are linked to the onset of inflammatory bowel disease (IBD). Conversely, other dietary patterns and specific food components can help protect or enhance the gut microbiota, contributing to the remission of IBD symptoms (22). Furthermore, the Mediterranean diet has also shown promising results in IBD. Studies have shown that Mediterranean diets increased diversity of microbiome composition, with a decreased concentration of *Firmicutes*, *Proteobacteria* and *Clostridia*, and an increase in *Bifidobacteria* and *Lactobacillus* (23) Increased diversity of microbiome composition, with a decreased concentration of *Firmicutes, Proteobacteria and Clostridia*, and an increase *in Bifidobacteria* and *Lactobacillus*. Another study presented the role of Mediterranean diet in promoting reduced inflammation levels, decreased cytokines release and a greater modulation on intestinal permeability (24). Additionally, vegan and vegetarian diets appear to promote the growth of beneficial microbial species during inflammatory states, such as *Bacteroides* and *Prevotella*, while reducing levels of *Bacteroides fragilis* and *Clostridium*. These diets also seem to trigger epigenetic changes that lower the risk factors for chronic inflammation (25).

The collective application of these microbial-based therapies highlighted a nuanced and promising approach to IBD treatment, opening new pathways through strategically manipulating the gut microbiome. The emergence of microbial-based therapies signals a transformative shift in the management of IBD, presenting options that are potentially safer and more effective. However, placing these therapies within the existing treatment framework is essential. Conventional pharmacological treatments, while effective in achieving remission and managing acute IBD episodes, are fraught with limitations and adverse effects that necessitate ongoing innovation and a deeper investigation into the gut microbiota’s healing potential. Historically, IBD management has focused on achieving remission and minimizing the severity of flares through pharmacological agents such as corticosteroids, amino salicylic acid derivatives, thiopurines, methotrexate, and anti-TNF-α inhibitors (26). While these treatments have marked a significant advancement in IBD care, there remains a subset of patients who either do not respond adequately, become resistant, or experience debilitating adverse effects, such as infectious and neoplastic complications (27). For instance, the long-term use of corticosteroids is associated with a spectrum of complications, including peptic ulcers, Cushing’s syndrome, osteoporosis, avascular necrosis, type 2 diabetes mellitus, and amenorrhea (28). These side effects carry a heightened risk of morbidity and mortality compared to biologic therapies and immunomodulators (29). Interestingly, the efficacy of certain treatments may be contingent upon the patient’s unique gut microbiome composition. For instance, sulfasalazine, a conventional treatment for IBD-associated peripheral spondylarthritis, has been recently shown to be effective in patients with a gut microbiome rich in *Faecalibacterium prausnitzii* and butyrate-producing bacteria (30). This correlation highlights the potential dependency of treatment efficacy on the composition of the gut microbiota, emphasizing the necessity for more in-depth research into its role. A deeper understanding of the interplay between the gut microbiota and IBD could pave the way for more effective and better-tolerated therapeutic strategies, steering away from the limitations of current treatments.

The early research has identified multiple strain of microbes with strong link to IBD particularly Crohn’s disease, have highlighted the role of certain strains of *Escherichia coli* (*E. coli*), such as Adherent and Invasive *E. coli* (AIEC). In addition to other species such as *clostridium difficile*, *Mycobacterium avium* subspecies *paratuberculosis* (MAP), and *Fusobacterium varium* (31). One of the pioneering studies that brought attention to AIEC, specifically strain LF82, was conducted in the early 2000s. It was found that ileal mucosa of CD patients is abnormally colonized by pathogenic *E. coli* strains termed AIEC for adherent-invasive *E. coli*. The study also demonstrated that LF82 could adhere and eventually invade the intestinal epithelial cells, a behavior that was not typical of other non-pathogenic *E. coli* strains. This discovery was crucial in understanding how certain bacterial strains could contribute to the pathology of IBD in humans (32). The study and analysis of the gut microbiome have evolved significantly over the years, employing various methods to understand the complex microbial communities in the gut. Historically, traditional microbial culturing was widely used to analyze gut samples; however, the limitation to this was the fact that many gut bacteria are not easily cultured under standard laboratory conditions, leading to an incomplete understanding of the microbiome (33). Furthermore, In the 1960s, Carl Woese began studying 16S ribosomal RNA (16S rRNA) genes, which encode part of the 30S small subunit of ribosomes in prokaryotes. These genes contain both highly conserved and variable regions, making them useful for accurately determining microbial phylogeny. Woese’s profiling of 16S rRNA genes across various bacteria revolutionized microbiology and established the field of molecular phylogenetics. This work also laid the groundwork for metagenomics—the study of genetic material recovered directly from environmental samples—enabling the exploration of uncultured microorganisms and their roles within microbial communities (34, 35).

In this study, we meticulously evaluated 71 studies to elucidate the impact of various nutraceutical interventions—namely, probiotics, prebiotics, synbiotics, and FMT—on patients with IBD. Our analysis encompasses a broad spectrum of outcome measures, including disease activity indices, inflammatory biomarkers, and serum cytokine levels, providing a comprehensive assessment of the therapeutic effects of these interventions. Furthermore, we scrutinize alterations in microbiome composition post-treatment, investigate the rates of remission and relapse across different therapeutic modalities, and assess the prevalence and severity of treatment-associated side effects. This thorough examination extends to exploring specific promising nutraceuticals, their optimal formulations, and identifying the optimal responder populations, aiming to determine their potential for personalized precision medicine. Conclusively, we synthesize the extant knowledge on FMT to present an updated perspective on its efficacy and therapeutic positioning in managing IBD.

## 2 Methods

### 2.1 Search strategy

This search was conducted in August 2023 by a health information professional using PubMed, Embase (OVID), Cochrane Library, Web of Science, and Scopus. The search involved utilizing the terms ‘gut microbiota’, ‘prebiotic’, ‘probiotic’, ‘symbiotic’, ‘fecal microbiota transplant’, and ‘inflammatory bowel diseases’, employing a combination of keyword terms and controlled vocabulary. Filters for language and year restrictions were not applied. Following the initial search, duplicate entries were identified and excluded to streamline the review process. Reference lists of published reviews were retrieved and scrutinized to locate additional studies. Complete details of the search methodology and parameters are documented in Appendix.

### 2.2 Title, abstract and full-text screening

Using the above search strategy, we imported all relevant papers into Covidence software (Melbourne, Australia). We then systematically reviewed the titles and abstracts, followed by a full text review conducted by at least two independent reviewers. A third reviewer resolved any conflicts. We included published human clinical trials in English, covering populations of any sex, ethnicity, and region, with patients diagnosed with IBD. We considered interventions such as probiotics, prebiotics, symbiotics, postbiotics, and FMT as interventions of interest. We excluded studies that were not in English, studies involving populations with diseases other than IBD, and other study types such as animal experiments, protocols, reviews, conference abstracts, etc.

### 2.3 Data extraction and analysis

Selected studies were extracted onto pre-piloted forms using Microsoft Excel 2016. The extracted parameters included first author, country, study type, sample size, age, contents, administration route, daily dose in both intervention and control groups, study duration, and outcome indicators. We processed the included articles for qualitative analysis, grouping relevant information by themes, patterns, and significance and expanding these through discussion.

## 3 Results

### 3.1 Data sources and search strategy

**Figure 1:**
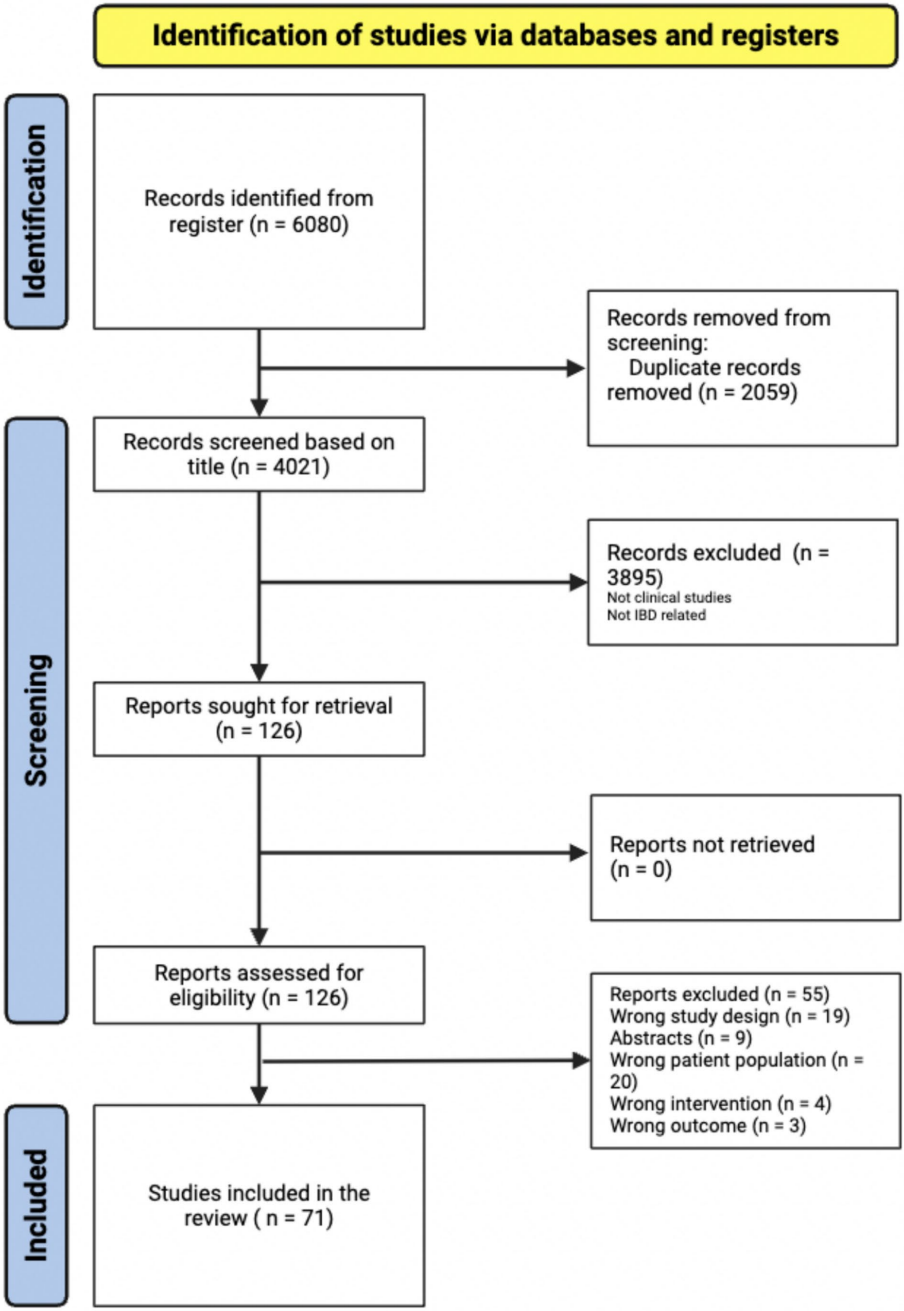
PRISMA flow chart of study selection.

The initial search yielded 6,080 items retrieved from PubMed, Web of Science, Scopus, and Google Scholar. After the automatic removal of 3,895 duplicates by Covidence, 4,021 articles were available for the title and abstract screening, of which 126 articles proceeded to full text screening. From this group, 71 articles were included in the systematic review (Figure 1).

### 3.2 Clinical trials characteristics

**Figure 2:**
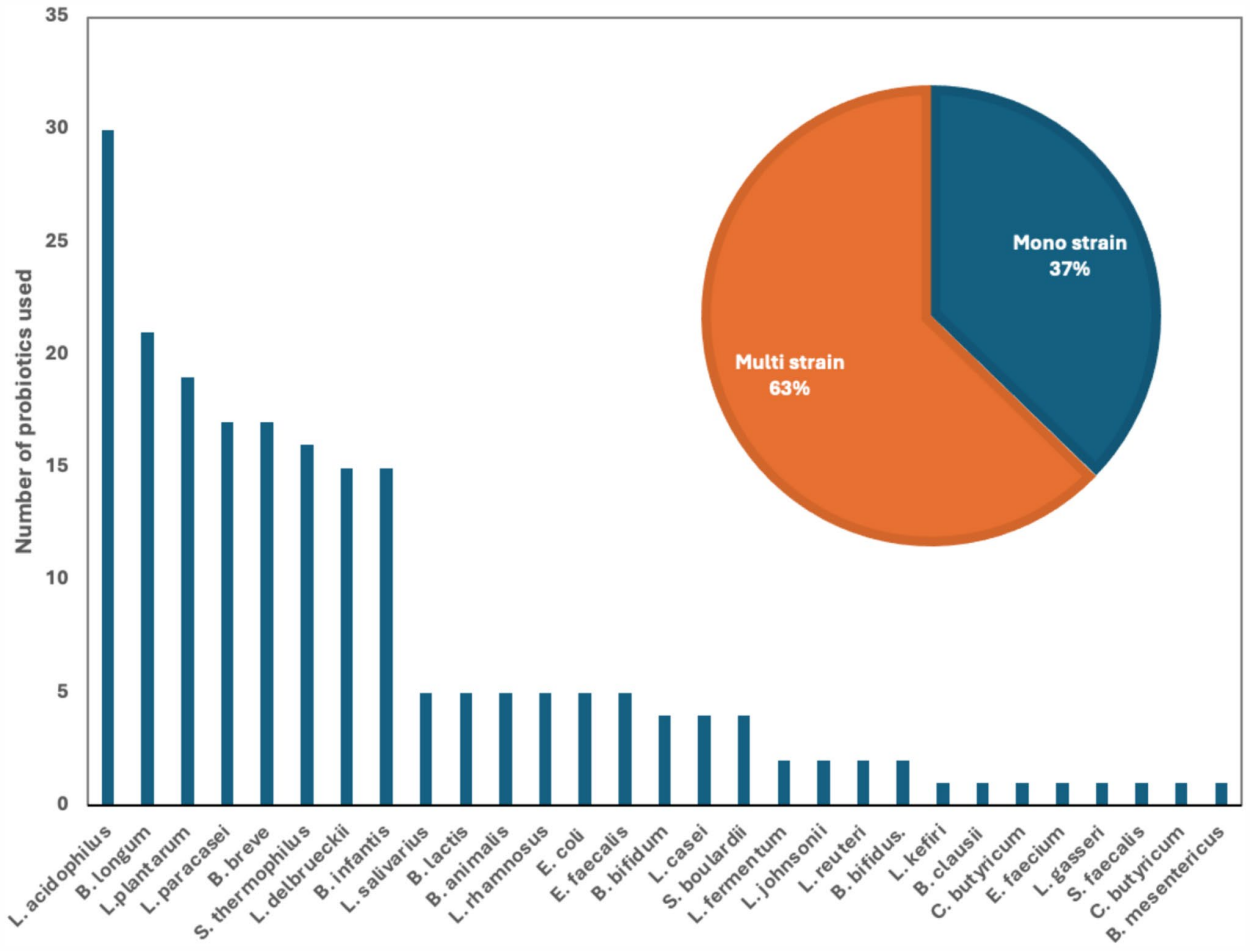
Number of used probiotics among reviewed clinical trials for patients with IBD. The figure shows that the most common probiotics used by different studies was *L. acidophilus* (14.5%) *B. longum* (10%) and *L. plantarum* (9%). The insert shows that 63% of the studies used multi strain comparing to 37% of the studies that used mono strain probiotic.

**Figure 3:**
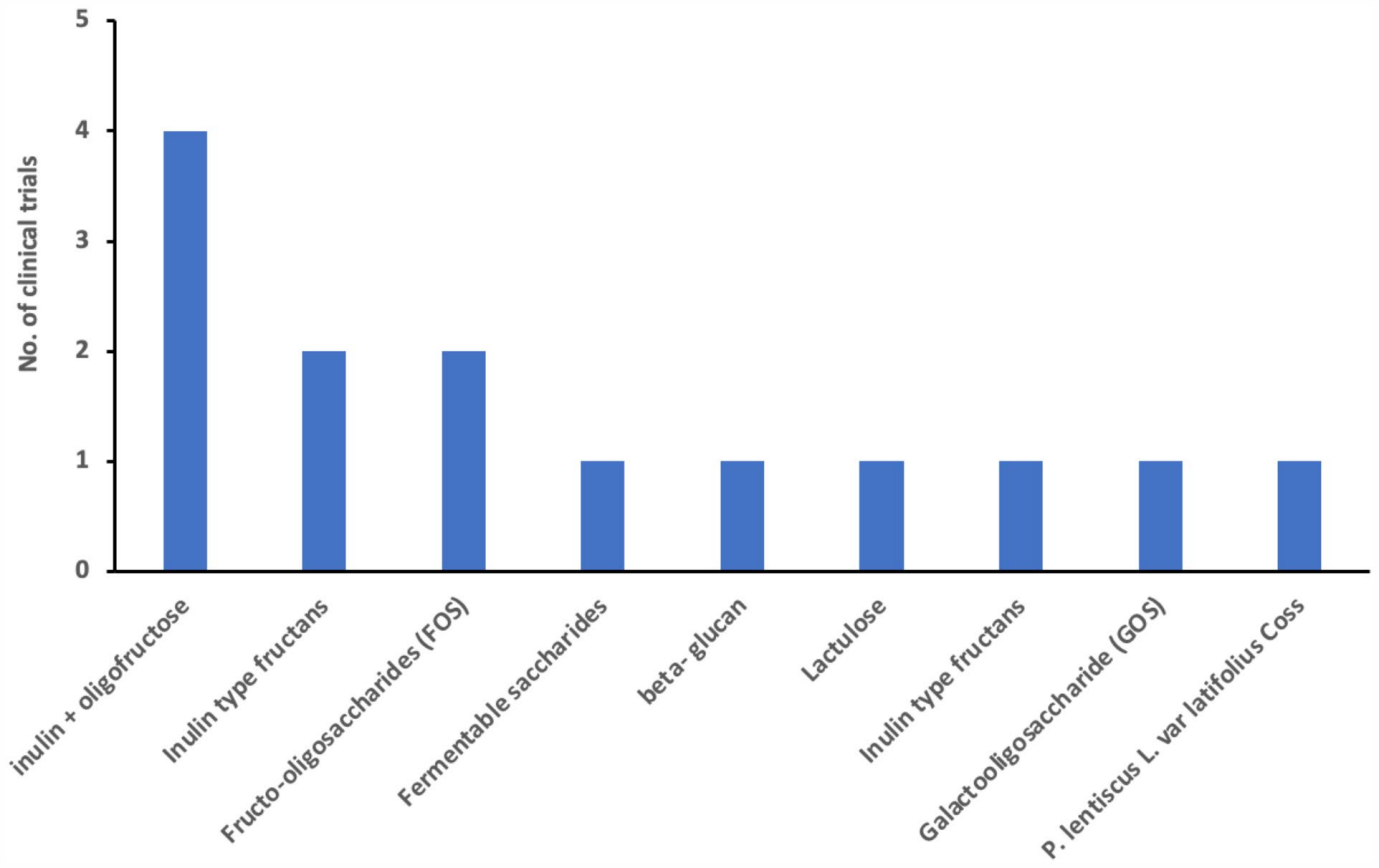
Frequency of prebiotics used among clinical trials for patients with IBD.

We examined 71 studies to analyze the characteristics of clinical trials focusing on interventions for IBD. This examination specifically included investigations into the efficacy of probiotics (Table 1), prebiotics (Table 2), synbiotics (Table 3), and FMT (Table 4). We found that the majority of interventions in the studies utilized probiotics (41 studies), followed by prebiotics (13 studies), FMT (11 studies), and synbiotics (6 studies), with no studies on postbiotics. The frequency of probiotic bacteria administrated in different trials is shown in Figure 2. Among the probiotic interventions, majority of the studies used multi strain probiotics. And the multi strain probiotic consisting of a mixture of eight bacterial strains, being the most used among the 41 studies. The most common probiotics used by different studies was *L. acidophilus*, *B. longum* and *L. plantarum* (Figure 2). In prebiotics, oligofructose-enriched inulin received the most attention, followed by inulin-type fructans (Figure 3). Most studies administered their interventions orally, while only two studies in the probiotic group involved rectal delivery. All the studies covered in this review encompassed a range of IBD subpopulations, predominantly involving patients with UC (Table 5).

**Table 1:** Studies investigating the effect of probiotics on various outcomes measures in inflammatory bowel disease.

Author Country Type of study	Intervention microbial composition	Intervention dose and duration	Control used and duration	Participants characteristics	N and age of participants	N_C_	N_I_	Primary outcome	Secondary outcome
Bamola et al. (42) India RDBPCT	*B. clausii*	2 billion CFU twice a day for 4 weeks	Maltodextrin excipient twice a day for 4 weeks	IBD	108 patients, aged 18–60 years	54	54	Microbiome composition (*Firmicutes* and anaerobic bacterial genera *Lactobacillus, Bifidobacterium and Faecalibacterium* were significantly increased in the intervention group)	Serum cytokines (significant decrease in TNF-α, IL-6, IL-1β in intervention group). No significant difference in CDAI score of patients with CD between groups. SCCAI score decreased significantly among patients with UC in the intervention group.
D’Incà et al. (104) India RCT	*L. casei*	First group (*n* = 8) 5-ASA (2.4 g/day) plus oral *L. casei* DG (8 × 10^8^ CFU) twice daily. Second group (*n* = 11) 5-ASA (2.4 g/day) plus rectal *L. casei* DG (8 × 10^8^ CFU) twice daily.	5-ASA 2.4 g/day oral	Mild left sided UC	26	8, 11	7	Microbiome composition (*Enterobacteriaceae* spp. increased significantly in the intervention group)	Serum cytokines and toll-like receptor expression (significant decrease in IL-1β and TNF-α mRNA mucosal levels, and significant increase in IL-10 mRNA in oral 5-ASA + rectal *L. casei* DG group)
Oliva et al. (41) Italy RPCT	*L. reuteri*	10 × 10^10^ CFU of *L. reuteri* ATCC 55730 enema for 8 weeks	Identical placebo	Ulcerative proctitis and/or proctosigmoiditis	31	15	16	Mayo Disease Activity Index (decreased significantly in the intervention group)	Serum cytokines (TNF-α and IL-1β significantly decreased in the intervention group)
Palumbo et al. (139) Italy RBDPCT	*L. salivarius, L. acidophilus B. bifidus.* BGN4	Mesalazine 1,200 mg and double daily probiotic blend for 2 years	Mesalazine 1,200 mg for 2 years	Moderate to severe UC	60	30	30	Modified Mayo Disease Activity Index (combination therapy showed better improvement vs. controls)	N/A
Shadnoush et al. (54) Iran RDCT	*L. acidophilus, B. animalis*	250 g of probiotic yogurt contained *Lactobacillus acidophilus* La-5 and Bifidobacterium BB-12	Placebo	IBD	210	105	105	Microbiome composition (mean numbers of *Lactobacillus, Bifidobacterium, and Bacteroides* significantly increased in the intervention group)	N/A
Tamaki et al. (59) Japan RDCT	*B. longum*	2–3 × 10^11^ freeze-dried viable BB536 for 8 weeks	Placebo	UC	56	28	28	Disease Activity Index (significant improvement of UCDAI scores in 8 weeks compared to control group)	N/A
Yılmaz et al. (148) Turkey Prospective open label RCT	*L. kefiri*	400 mL/day kefir for 4 weeks	Control mentioned but was not received	IBD	45	20	25	Inflammation and quality of life (Significant decrease in ESR and CRP in test group and improved quality of life)	N/A
Yasueda et al. (147) India RCT	*C. butyricum*	Nine tablets of MIYA-BM for 24 months	Placebo	UC	17	9	8	Microbiome composition (numbers of the *Escherichia* were significantly decreased in the intervention group)	N/A
Bjarnason et al. (56) London RDBPCT	*L. rhamnosus, L. plantarum, L. acidophilus, E. faecium*	Symprove 1 mL/kg/day for 4 weeks	Identical placebo (1 mL/kg/day) for 4 weeks	Asymptomatic IBD	143 patients, age 18–70	70	73	Quality of Life Questionnaire results (QOL) (no significant differences in IBD-QOL scores between both groups)	Disease activity and lab values (fcal, ESR, CRP) (no significant difference in all parameters except for fcal significantly deceased in UC patients)
Garcia Vilela et al. (133) Brazil RCT	*S. boulardii*	*S. boulardii* every 8 h as an oral capsule formulation which contained 200 mg lyophilized *S. boulardii*-17 (about 4,108 cells), 6 mg sucrose and 2.4 mg magnesium stearate (Floratil†).	Placebo every 8 h as a capsule containing 200 mg cellulose, 6 mg sucrose and 2.4 mg magnesium stearate	Remission CD	34 patients age range 19–54 years, mean 37 years	19	15	Intestinal permeability was improved in the intervention group	N/A
Sood et al. (57) India RDBPCT	*L. paracasei, L. plantarum, L. acidophilus, L. delbrueckii, B. longum, B. breve, B. infantis, S. thermophilus*	Probiotic mixture twice daily for 12 weeks. Four sachets of were given daily equating to a dose of 3,600 billion viable lyophilized bacteria.	Identical placebo sachets containing maize powder and taken mixed with water or yogurt twice daily for 12 weeks.	Mild-to-moderately active UC	84 Adult patients >18 y	29	55	Disease Activity Index (significant improvement in UCDAI in the intervention group)	Remission rate (remission was achieved in the intervention group, difference was significant)
Kato et al. (48) Japan RPCT	*B. breve, B. bifidum, L. acidophillus*	Bifidobacteria-fermented milk 100 mL each day for 12 weeks	Identical placebo for 12 weeks	Active UC	20	10	10	Disease Activity Index (significant improvement in CAI score in the intervention group)	Microbiome composition (numbers of *B. breve* and *B. pseudocatenulatum* among Bifidobacterial species were significantly increased in the BFM group)
Kruis (58) Germany RDBDDT	*E. coli*	*Escherichia coli* of strain Nissle 1917 (serotype O6:K5:H1) (Mutaflor 100 mg) test group received one capsule of Mutaflor 100 mg once daily and one tablet of placebo three times daily from day 1 to day 4, and two capsules of Mutaflor 100 mg once daily and one tablet of placebo three times daily from day 5 to the end of the study.	Mesalazine, consisting of eudragit L coated 5-aminosalicylic, 5-ASA, 5-aminosalicylic acid (Salofalk500 mg). Control group received one capsule of placebo once daily and one tablet of Salofalk 500 mg three times daily from day 1 to day 4, and two capsules of placebo once daily and one tablet of Salofalk 500 mg three times daily from day 5 to the end of the study 12 months	Remission UC	327 patients, aged 18–70 years	165	162	Relapse rate (no significant difference in number of patients who relapsed in both groups)	Quality of life and endoscopic and histological analysis (no significant change in both groups)
Cui et al. (130) China RCT	*B. longum, L. acidophilus, E. faecalis.*	Bifid triple viable capsule (BIFICO) (1.26 g/d), for 8 wk.	Identical placebo (starch) for 8 wk.	Active UC	30 patients, aged	15	15	Microbiome composition (number of Gram-positive Bacillui and Enterococci was significantly higher in the intervention group)	Cytokines and inhibitive factor detection (expressions of NF-κB p65 and DNA binding activity of NF-κB were significantly decreased and anti-inflammatory cytokines were increased)
Miele et al. (105) Italy Prospective single center DBPCT	*L. paracasei, L. plantarum, L. acidophilus, L. delbrueckii, B. longum, B. breve, B. infantis, S. thermophilus*	Probiotic mixture 900 billion viable lyophilized bacteria provided in packets instructed to put in in cold water or any noncarbonated drink once daily + daily mesalazine for 1 year	Identical placebo contained 3 g of corn starch associated to concomitant steroid induction and oral mesalamine maintenance treatment once daily + daily mesalazine for 1 year	Newly diagnosed with UC	29 patients, mean age: 9.8 years; range: 1.7–16.1 years	15	14	Disease Activity Index (not significant differences between intervention and placebo patients in Lichtiger colitis activity index)	Remission rate (remission was achieved in 13 patients (92.8%) treated with VSL#3 and the difference was significant compared to controls)
Hegazy (106) Egypt RCT	*L. delbruekii, L. fermentum*	Probiotic + sulfasalazine 2,400 mg/day for 8 weeks	Placebo (starch) + sulfasalazine 2,400 mg/day for 8 weeks	UC patients with chronic diarrhea	30 patients	15	15	Endoscopic and histological (Administration of probiotic plus oral sulfasalazine inhibited the extent of inflammation, prevented mucosal injury, and alleviated colitis)	Serum cytokines + Fcal (IL-6+ fcal decrease in the intervention was significant compared to controls)
Matthes et al. (96) Germany Explorative, MC, parallel groups RDBPCT	*E. coli*	Enema. EcN 40 mL, EcN 20 mL, EcN 10 containing 10E8 EcN/ml once daily for 2 weeks	Identical placebo enema once daily for 2 weeks	Diagnosis of acute UC proctitis/proctosigmoiditis	90 patients between 18 and 70 years	20	24, 23, 23	Remission rate (remission rate was dose dependent Time to remission was shorter in the 40 mL and 20 mL EcN groups than in the 10 mL EcN and control groups)	N/A
Ballini et al. (128) Italy RDBPCT	*L. plantarum, L. fermentum, L. acidophilus, B. infantis, L. casei, B. longum, L. rhamnosus, B. lactis, L. reuteri, L. salivarius, L. paracasei, L. gasseri, B. bifidum, B. breve, S. thermophilus.*	Hyperbiotic PRO-15 probiotic three per day for the first month and one tablet per day for the remaining 2 months (total 3 months)	Identical placebo three per day for the first month and one tablet per day for the remaining two months (totals 3 months)	IBD	40 patients aged 30–60 years	20	20	Oxidant ability (d-ROMs test) and for the antioxidant response (BAP test) (overall improvement in oxidative stress)	N/A
Li et al. (70) China RCT	*B. longum, L. acidophilus, E. faecalis.*	Bifid Triple Viable (2 capsules three times daily) and 5-aminosalicylic acid (1 g two times daily) for 8 weeks	5-aminosalicylic acid (1 g two times daily) for 8 weeks	Active UC	82 patients, aged 21–70	41	41	Disease Activity Index (Mayo scores were significantly lower in the intervention group)	N/A
Ng et al. (137) UK DBPCT	*L. paracasei, L. plantarum, L. acidophilus, L. delbrueckii, B. longum, B. breve, B. infantis, S. thermophilus*	2 sachets twice/day of the probiotic mixture (3,600 billion bacteria), for 8 weeks.	Identical placebo containing maize starch 2 sachets twice/day for 8 weeks.	Mild to moderately active UC	28	14	14	Effect on dendritic cell cytokines (DC TLR-2 expression decreased, IL-10 production increased and IL-12p40 production decreased)	N/A
Yoshimatsu et al. (107) Japan RDBPCT	*S. faecalis, C. butyricum, B. mesentericus*	2 mg, three tablets/day for 1 year	Identical placebo t.i.d/1y	Inactive UC	46 patients	23	23	Relapse rate (relapse rates were significantly higher in the intervention group)	Remission rate (may be effective for maintaining clinical remission in patients with quiescent UC)
Matsuoka et al. (50) Japan RDBPCT	*B. breve, L. acidophilus*	One pack of *B. breve* strain Yakult fermented milk (Mil–Mil) per day for 48 weeks	Placebo (one pack of energy beverage) per day for 48 weeks.	UC	195 patients, aged 20–70 years	97	98	Relapse rate (no difference between both groups)	Disease activity index + fecal microbiota (no significant differences in the mean DAI scores + significant decrease in Bifidobacterium species)
Wildt et al. (108) Denmark RDBPCT	*L. acidophilus, B. animalis*	Two capsules three times daily, resulting in a total delivery of 1.5 × 10^11^ CFU daily for 52 weeks	Identical placebo two capsules three times daily for 52 weeks	Left-sided ulcerative colitis in remission	32 patients, aged 23–68	12	20	Remission and relapse rate (no significant difference between both groups)	Safe and well tolerated
Fan et al. (40) China RCT	*B. longum, L. acidophilus, E. faecalis.*	2 or 6 (Bifico) + 1, 2, 3 or 6 (Pentasa) tablets	Pentasa (mesalazine extended action tablet) 1, 2, 3 or 6 tablets	IBD	40 patients >18	19	21	Relapse rate (no significant difference both groups)	Microbiome composition + inflammatory markers (number of bifidobacteria and lactobacilli was significantly increased, while enterobacteria, enterococci, saccharomyces, and bacteroides decreased significantly + CRP levels were significantly lower in treatment group)
Huang et al. (109) China RCT	*B. longum, L. acidophilus, E. faecalis.*	BTV capsules + mesalazine (1.26 g + 3 g) two capsules of BTV before meals 3times/day + four tablets before meal, 3 time/d of mesalazine	Mesalazine 3 g four tablets oral administration before meal, 3 time/d for 8 weeks	UC	360 patients aged >18	180	180	Disease activity index + remission rate (UCDAI and clinical symptoms scores improved and treatment was effective in remission induction)	Serum cytokines (IL-10 and IL-8 both were deceased in the intervention group)
Su et al. (110) China RCT	*B. longum, L. acidophilus, E. faecalis.*	Probiotics: Bifidobacterium Lactobacillus triple tablets + glucocorticoides 4 g + 4 g + 0.75–1.0 mg/kg/day and gradually stopped in 3–4 months	Sulfasalazine 4 g/day	CD	83 patients aged >18	40	43	Clinical efficacy + microbiome composition (therapeutic efficiency of the treatment group was significantly higher) (levels of yeast, enterococci and peptococcus of the two groups of patients were significantly decreased, while the level of lactobacillus was significantly increased in the intervention group)	Serum cytokines (TNF-α and IL-10 were significantly decreased in both groups)
Petersen et al. (111) Denmark RDBPCT	*E. coli*	Ciprofloxacin and probiotic *Escherichia coli* Nissle	Placebo + Ciprofloxacin	Active UC	100 patients >18	50	50	Remission rate (fewer patients achieving remission in the intervention group)	N/A
Bourreille et al. (112) France RDBPCT	*S. boulardii*	*S. boulardii* treatment at a daily dose of 1 g for 52 weeks	Identical placebo 1 g for 52 weeks	Remission CD	159 patients >18	79	80	Relapse rate (median time to relapse did not differ significantly between patients given *S. boulardii* vs. control)	Disease Activity Index (no significant differences between groups in mean CDAI)
Tursi et al. (144) Italy Multicenter, RDBPCT	*L. paracasei, L. plantarum, L. acidophilus, L. delbrueckii, B. longum, B. breve, B. infantis, S. thermophilus*	3.6 × 10^12^ CFU/day for 8 weeks	Standard pharmaceutical therapy (5-ASA and/or immunosuppressant) for 8 weeks	Mild to moderately active UC	144 patients >18	73	71	Remission rate (Remission was higher in the intervention group than in the control group)	Disease Activity Index (UCDAI scores decreased in the intervention group)
Tursi et al. (55) Italy Multicenter, RDBPCT parallel study	*L. paracasei, L. plantarum, L. acidophilus, L. delbrueckii, B. longum, B. breve, B. infantis, S. thermophilus*	750 mg of balsalazide + 3 g probiotic mixture for 8 weeks	A group with 4.50 g Balsalazide and another 2.4 g mesalazine for 8 weeks	Mild to moderately active UC	90 patients	30	30 + 30	Remission rate (Remission was higher in the intervention group than in the control group)	N/A
Zocco et al. (113) Italy Prospective, open-label, randomized trial	*L. rhamnosus*	Lactobacillus GG 18 × 10^9^ viable bacteria/day divided into two oral administrations (LGG group, 65 patients) OR Lactobacillus GG 18 × 10^9^ viable bacteria/day plus mesalazine 2,400 mg daily (LGG **+** MES group, 62 patients) OR	Mesalazine 800 mg tablets (three tablets 2,400 mg) daily (MES group, 60 patients)	Remission UC	187 patients	60	65 + 62	Relapse rate (no difference in relapse rate between groups)	Disease Activity Index (no statistically significant differences were reported using CAI)
Schultz et al. (114) USA RPCT	*L. rhamnosus*	2 × 10^9^ CFU/day for 6 months	Placebo for 6 months	Moderate–active CD	11	6	5	Relapse rate (no difference in relapse rate between groups)	N/A
Guslandi et al. (115) Italy RCT	*S. boulardii*	*S. boulardii* + Pentasa (mesalazine) 500 mg + 1 g for 6 months	Pentasa (mesalazine) 1.5 g for 6 months	CD in remission	32 patients aged 23–49	16	16	Relapse rate (intervention was effective in relapse rate; the difference is statistically significant)	N/A
Rembacken et al. (116) UK Single center RCT double dummy study	*E. coli*	A dose of two capsules twice daily (2.5 × 10^10^ viable bacteria per capsule) for 12 months	Mesalazine 2.4 g for 12 months	Active UC	116 patients aged 18–80	59	57	Remission rate + relapse rate (intervention was not significantly different from control)	N/A
Kruis (136) Germany Single center RCT double dummy study	*E. coli*	1–4 days: 2.5 × 10^10^ viable bacteria, the rest of period: 5 × 10^10^ viable bacteria EcN + mesalazine for 12 weeks	500 mg mesalazine + identical placebo to EcN for 12 weeks	Inactive UC	103 patients >17 years	53	50	Relapse rate (intervention was not significantly different from control)	Disease Activity Index (no statistically significant differences were observed using CAI)
Pavel et al. (140) India Prospective comparative study	*S. boulardii*	*S. boulardii* 1 g daily for six months	–	CD	49 patients	28	21	Microbiome composition (Escherichia and Enterobacter spp. decreased significantly. *Faecalibacterium prausnitzii* Bifidobacterium spp. and Bacteroidesspp. increased significantly)	N/A
Huynh et al. (51) Canada Open label study	*L. paracasei, L. plantarum, L. acidophilus, L. delbrueckii, B. longum, B. breve, B. infantis, S. thermophilus*	Multi strain probiotic mixture	N/A	Mild to moderate UC	18 patients aged 3–17 mean age 12	18	N/A	Disease Activity Index (significant decrease in SCCAI)	Safe and well tolerated
Chen et al. (95) China RDBPCT pilot study	*L. casei, L. plantarum, B. animalis*	Two sachets of probiotic product for 12 weeks	Placebo two sachets of probiotic product for 12 weeks	Active UC	25 patients	13	12	Microbiome composition (significantly more beneficial bacteria like *Eubacterium ramulus Pediococcus pentosaceus Bacteroides fragilis* and *Weissella cibaria*)	Disease Activity Index (significant decrease in the intervention)
Dore et al. (131) Italy Retrospective cohort study	*L. paracasei, L. plantarum, L. acidophilus, L. delbrueckii, B. longum, B. breve, B. infantis, S. thermophilus*	Probiotic 108 CFU 1 tablet per day for 1 week	–	IBD	200 patients mean age of 39.7 ± 15.2 years	N/A	200	Safety and side effects (reduction in the number of total adverse events in those taking probiotic for ≥75% of disease course was more evident in UC patients than in CD patients)	N/A
Bibiloni, et al. (2005) Canada Open label study	*L. paracasei, L. plantarum, L. acidophilus, L. delbrueckii, B. longum, B. breve, B. infantis, S. thermophilus*	Probiotic mixture 3,600 billion bacteria daily in two divided doses for 6 wk.	N/A	Active UC	32 patients aged 18–65 yr	N/A	32	Remission rate (intervention resulted in induction of remission/response rate of 77%)	N/A
Shadnoush et al. (53) Iran RBDPCT	*L. acidophilus, B. animalis*	250 g of probiotic yogurt contained *Lactobacillus acidophilus* La-5 and Bifidobacterium BB-12. 106 colony forming units (CFU) per each gram of yogurt. Daily for 8 weeks	Plain yogurt daily for 8 weeks	IBD	210 patients aged 26–59 year. Mean age 37.69	90	86	Serum cytokines (decreased serum levels of IL-1b, TNF-a and CRP but increased the serum levels of IL-6 and IL-10)	Microbiome composition (mean numbers of Lactobacillus, Bifidobacterium, and Bacteroides in intervention group were significantly increased)

RCT, randomized clinical trials; RDBPCT, randomized double-blind placebo controlled clinical trial; MC, multicenter; UC, ulcerative colitis; CD, Crohn’s disease; IPAA, ileal pouch-anal anastomosis; FMT, fecal microbiota transplant; CFU, colony forming units; FOS, fructooligosaccharides; GOS, galactooligosaccharides; CAI, clinical activity index; UDCAI, Ulcerative disease activity index; CDAI, Crohn’s disease activity index; SCCAI, simple clinical colitis activity index; HBI, Harvey-Bradshaw index; MMDAI, Modified Mayo disease activity index; CRP, C-reactive protein; ESR, erythrocyte sedimentation rate; Fcal, fecal calprotectin; TNF-α, tumor necrosis factor-alpha; N/A, not applicable; N/R, not reported. Most modes of administration were oral; a few rectal/enema interventions are specified.

**Table 2:** Studies investigating the effect of prebiotics on various outcomes measures in inflammatory bowel disease.

Author	Country	Type of study	Intervention	Route of administration	Intervention dose and duration	Control duration of therapy	Participant characteristics	N and mean age (SD)	N, control group	N, intervention group	Primary outcome	Secondary outcome	Clinical benefit
Benjamin et al. (117)	UK	RDBPCT	prebiotic-fructo-oligosaccharides (FOS)	Oral	15 g/day for 4 weeks	Placebo 15 g for 4 weeks	Active CD	103 patients >18 years	49	54	Disease Activity Index (no significant difference in the mean CDAI between the FOS and placebo groups after treatment)	Remission rate and fecal microbiota (no significant difference in the numbers achieving clinical remission between groups FOS vs. placebo)	No
Halmos et al. (135)	India	randomized, controlled cross-over study	Prebiotics: fermentable oligosaccharides, disaccharides, monosaccharides and polyols	Oral	Low FODMAPs diet for 21 days	Australian diet for 21 days	Quiescent Crohn’s disease	16	8	8	Fecal microbiota (relative butyrate-producing Clostridium cluster XIVa abundance was higher in the intervention group)	n/a	Yes
Ikegami et al. (118)	Japan	RCT	Prebiotic-1-ketose (FOS)	Oral	10 g/day for 8 weeks	Maltose	Mild to moderate UC	37 patients aged 20–80 years	19	18	Disease Activity Index (CAI was significantly lower in the 1-kestose group than in the placebo group)	Remission rate and fecal microbiota (Clinical remission and response rates were higher in the 1-kestose group + decreased relative abundance of several bacteria, including Ruminococcin gnavus group)	Yes
Papada et al. (49)	Greece	RDBPCT	Prebiotics-oral mastiha (*Pistacia lentiscus*)	Oral	2.8 g/day for 3 months	Identical placebo tablets for 3 months	IBD	60 patients 18–67 years	27	33	Disease Activity Index and quality of life questionnaire (Significant decrease in HBI)	N/A	No
Hafer et al. (134)	Germany	RCT pilot study	Prebiotics - Lactulose	oral	15 mL lactulose syrup (containing 10 g lactulose) daily for 4 months	standard pharmaceutical therapy for 4 months	IBD	31	17	15	Disease Activity Index (no significant improvement in CAI)	Quality of life (Significant improvement)	No
Casellas et al. (119)	Spain	RDBPCT pilot study	prebiotics – Oligofructose + inulin	oral	Oligofructose-enriched inulin 12 g for 2 weeks	placebo 12 g for 2 weeks	active UC	19 patients aged 18–75 years	10	9	Disease Activity Index (Rachmilewitz score decreased in both groups)	Inflammatory markers (early significant reduction of calprotectin was observed in the group receiving oligofructose-enriched inulin)	Yes
De Preter et al. (120)	Belgium	DBRCT	prebiotics- oligofructose + inulin	oral	OF-IN (ORAFTISynergy-1) 1:1 mixture of inulin and oligofructose 20 g/day for 4 weeks	Placebo (Maltodextrin) 20 g/day for 4 weeks	Inactive, moderate active CD	45 patients, aged >18	20	25	Fecal SCFAs (In patients receiving OF-IN, the relative levels of acetaldehyde and butyrate were significantly increased)	N/A	Yes
Joossens et al. (121)	Belgium	DBRCT	prebiotics- oligofructose + inulin	oral	Oligofructose-enriched inulin (OF-IN) 20 g/day for 4 weeks	Placebo 20 g/day for 4 weeks	Inactive, moderate active CD	45 patients	20	25	Fecal microbiota (significant increase in the number of *B. longum* was found, whereas *R. gnavus* decreased after OF-IN intake)	N/A	Yes
Anderson et al. (122)	UK	case contol study	prebiotic - inulin type fructans	Oral	Interviewer-administered questionnaires to measure intakes of inulin-type fructans from habitual diet in patients with active Crohn’s disease, inactive Crohn’s disease and healthy controls	NR	Active CD/inactive CD/healthy controls	98/99/106	106	197	Measure intake of inulin type fuctans between the groups (Patients with active Crohn’s disease consume lower quantities of fructans and oligofructose than their inactive counterparts and healthy controls)	Association between intake and disease activity (Harvey–Bradshaw Index, HBI) negative associations were found	No
Hedin et al. (123)	UK	RCT	Prebiotic – inulin + oligofructose	Oral	Oligofructose/inulin (15 g/day) for three weeks	NR	CD	Aged between 16 and 35 years	12	19	Inflammatory markers (no significant change in Fca)	Microbiome composition (Faecal Bifidobacteria and *Bifidobacterium longum* increased in patients and siblings; *Bifidobacterium adolescentis* and Roseburia spp. increased only in siblings. Compared with patients, siblings had a greater magnitude change in Bifidobacteria)	No
Nyman et al. (138)	Sweden	RCT	Prebiotics - oat bran (beta- glucan)	Oral	60 g of oat bran corresponding to an intake of 12 g dietary fiber (consistent to 6 g of β-glucan) for 24 weeks	low-fiber wheat products (providing 5 g dietary fiber daily and <0.5 g β-glucan) for 24 weeks	UC	200	63	67	Fecal SCFAs (higher fecal butyrate concentrations and lower serum LDL level)	N/A	Yes
Valcheva et al. (124)	Canada	RCT pilot study	Prebiotics - inulin-type fructans	Oral	15 g (*n* = 13) daily oral oligofructose-enriched inulin (Orafti®Synergy1) for 9 weeks.	7.5 g (*n* = 12) daily oral oligofructose-enriched inulin (Orafti®Synergy1) for 9 weeks	Mild-to-moderate UC	25 patients aged 18–65 years	12	13	Disease Activity Index (significantly decreased activity index)	Microbiome composition (Increased Bifidobacteriaceae and Lachnospiraceae abundance)	Yes
Wilson et al. (125)	UK	Open label study	Prebiotics - galactooligosaccharide (GOS)	Oral	2.8 g/d GOS for 6 weeks.	NA	Mildly active UC	13 patients aged 16–65 years	NA	13	Immune-related gene expression (Five genes were upregulated and two downregulated)	Microbiome composition and inflammatory markers (Bifidobacterium and Christensenellaceae proportions only increased in patients with less active diseases SCCAI less than 2)	No

**Table 3:** Studies investigating the effect of synbiotics on various outcomes measures in inflammatory bowel disease.

Author	Country	Type of study	Type of intervention	Route of administration	Intervention dose and duration	Control and duration	Participant characteristics	N and mean age (SD) of participants	N, control group	N, intervention group	Primary outcome	Secondary outcome	Clinical benefit
Ishikawa et al. (52)	Japan	RCT	Synbiotic - Bifidobacterium (*Bifidobacterium breve* strain Yakult, BbY) as probiotic + galacto-oligosaccharide (GOS) as prebiotic	Oral	1 gram of the freeze-dried powder containing probiotic BbY (10^9^ CFU/g) was administered immediately after every meal thrice a day, and 5.5 g of GOS were administered once a day for a year	Appropriate medical treatment of UC (salazosulfapyrdidine, mesalazine, steroids)	Active UC	41 patients	20	21	Endoscopic score (endoscopic score of the intervention group was significantly lower than that of the control group)	Microbiome composition (Significant differences were seen in fecal number of Bacteroidaceae)	Yes
Steed et al. (43)	Scotland	RDBPCT	Synbiotic - *Bifidobacterium longum* and Synergy 1	Oral	1 capsule twice daily for 6 months	Identical placebo twice daily for 6 months	Active CD	24 patients age 18–79	11	13	Microbiome composition (significant increases in bifidobacteria and Enterococcal numbers increased at 3 and 6 months in the synbiotic group)	Disease Activity Index (significant improvement in CDAI scorers in the treatment group)	Yes
Amiriani et al. (44)	Iran	RDBPCT	Synbiotic - Lactocare (*Lactobacillus rhamnosus*, *Lactobacillus casei*, *Lactobacillus acidophilus*, *Bifidobacterium breve*, *Lactobacillus bulgaricus*, *Bifidobacterium longum*, *Streptococcus thermophilus*) + Fructooligosaccharides (FOS)	Oral	Lactocare® 1 capsule twice a day for 8 weeks	Identical palcebo containing starch for 8 weeks	Mild to moderate UC	60 patients, adult age 18–60	32	28	Disease Activity Index (significant improvement in SCCAI)	Inflammatory markers (no significant change in fcal)	Yes
Kamarli Altun et al. (126)	Turkey	RPCT	Synbiotic- six probiotic strains (3 × 109 CFU)-*Enterococcus faecium*, *Lactobacillus plantarum*, *Streptococcus thermophilus*, *Bifidobacterium lactis*, *Lactobacillus acidophilus*, *Bifidobacterium longum*-and prebiotic fructooligosaccharide (225 mg/tablet)	Oral	One table twice a day for 8 weeks	Identical placebo for 8 weeks	Mild-to-moderate UC	40 patients, aged >18	20	20	Acute phase reactants and clinical and endoscopic activities of the disease (improvement in the clinical activity was significantly higher in the synbiotic group)	N/A	Yes
Furrie et al. (132)	UK	RCT	Synbiotic - a probiotic, *Bifidobacterium longum*, isolated from healthy rectal epithelium, and a prebiotic (Synergy 1), a preferential inulin and oligofructose growth substrate	Oral	4 × 10^11^ freeze dried viable + 12 g for 4 weeks	Placebo (FOS + inulin) 12 g for 4 weeks	Active UC	18 patients, aged >18	9	9	Sigmoidoscopy scores (reduced inflammation and regeneration of epithelial tissue in the intervention group)	Serum cytokines (Tumor necrosis factor a and interleukin 1a were significantly reduced in treatment group)	Yes
Bousvaros et al. (127)	USA	RCT	Synbiotic - LGG + inulin	Oral	LGG, 1 capsule (containing at least 1,010 bacteria and 295 mg inulin) twice a day	Identical capsule containing 355 mg inulin (placebo).	Patients w small bowel, colonic, or perianal CD in remission	75 patients age 5–21	36	39	Microbiome composition (no significant difference between both group in the amount of lactobacillus)	Relapse rate (no significant difference on time of relapse between both groups)	No

**Table 4:** Studies investigating the effect of FMT on various outcomes measures in IBD.

Author	Country	Type of study	Route of administration	Donor	Intervention dose and duration	Control and duration	Bowel lavage	Participants characteristics	N and mean age (SD)	N, control group	N, intervention group	Primary outcome	Secondary outcome	Clinical benefit
Costello et al. (129)	Australia	RDBPCT	Enema	Multidonor FMT (3–4 unrelated donors)	300 mL of anaerobically prepared pooled donor FMT for 8 weeks	Autologous FMT 200 mL of fecal suspension, and a following 100 mL of fecal suspension	Yes	Mild-to-moderately active UC	73	35	38	Steroid free remission (the intervention was effective in inducing remission)	Clinical response (achieved significant in the intervention group)	Yes
Wang et al. (146)	China	Open-label, prospective clinical trial	Enema	Single donor FMT	Three infusions with an interval of 2–3 months	N/A	N/R	Active UC	16	N/A	16	Clinical response (clinical remission was achieved in the intevention group)	Serum cytokines (IL-1Ra, IL-6, IP-10 and ENA-78 decreased significantly after the second FMT)	Yes
Paramsothy et al. (63)	Australia	RDBPCT	Colonoscopy followed by enema	Multidonor FMT (3–7 unrelated donors)	Colonoscopic infusion followed by enemas 5 d/wk. for 8 weeks.	Placebo enema 5 d/wk. for 8 weeks	No	Active UC	81	40	41	Microbiome composition (*Eubacterium hallii* and Roseburia inulivorans increased in the intervention group)	N/A	Yes
Moayyedi et al. (60)	Canada	RDBPCT	Enema	Single donor FMT- unrelated	50 g stool in 50 mL saline infusion once weekly for 6 weeks	50 mL water enema once weekly for 6 weeks	Yes	Active UC	75	37	38	Clinical remission (clinical and endoscopic emission was achieved significantly in the intervention group)	N/A	Yes
Rossen et al. (61)	Netherlands	RDBPCT	Nasoduodenal tube	Donor FMT (related + unrelated)	Minimum 60 g stool in 500 mL of healthy donor FMT two infusions at week 1 and 3	Autologous FMT two infusions at week 1 and 3	Yes	Mild-to-moderately active UC	48	25	23	Clinical remission (no change in clinical remission after treatment)	N/A	No
Paramsothy et al. (99)	Australia	RDBPCT	Colonoscopy followed by enema	Multidonor FMT (3–7 unrelated donors)	37.5 g stool in 150 mL saline infusion 5d/week for 8 weeks	Placebo enema 5 d/wk. for 8 weeks	Yes	Active UC	81	40	41	Clinical and endoscopic remission (both achieved significantly in the intervention group)	Fecal microbiota	Yes
Sood et al. (142)	India	Pilot RCT	Colonscopic infusions	Single donor FMT	Colonoscopic infusion every 8 weeks for 48 weeks	Placebo infusions every 8 weeks for 48 weeks	Yes	Inactive UC	61	30	31	Clinical remission (no change)	Endoscopic and histologic remission (achieved significantly in the intervention group)	No
Březina et al. (62)	Prague	RCT	Enema	Single donor FMT	10 infusions (5 times in the first week then once weekly for 5 weeks)	4 g mesalamine enemas daily for 2 weeks and then every other day until the end of week 6	No	Mild-to-moderate left sided UC	43	22	21	Clinical remission (effective in inducing remission in the intervention group)	Microbiome composition (Increased microbial diversity persisted 3 months after FMT)	Yes
Sokol et al. (141)	France	Pilot RCT	Colonscopic infusions	Single donor FMT	50–100 g of stool resuspended in 250–350 mL of sterile sodium chloride once for 24 weeks	Placebo infusionson once for 24 weeks	No	Colonic or ileo-colonic CD	17	9	8	Successful colonization of the donor microbiota at 6 weeks (no change)	N/A	No
Suskind et al. (143)	US	Open-label, prospective clinical trial	Nasogastric tube	Single donor FMT	100 g of feces/100 or 200 mL of saline; one dose	N/A	No	Mild-to-moderately active CD	9	N/A	9	Disease activity index (improved in the intervention group)	N/A	Yes
Vaughn et al. (145)	US	Open-label, prospective clinical trial	Colonoscopy	Single donor FMT	50 g of feces/250 mL of saline; one dose over 12 weeks	N/A	Yes	CD	19	N/A	19	Clinical remission (achieved in the intervention group)	N/A	Yes

**Table 5:** Distribution of study patients according to primary disease.

Disease distribution	Number of studies	%
IBD	10	14.08
Active UC	38	53.51
Active CD	10	14.08
UC in remission	4	5.63
CD in remission	5	7.04
Ulcerative proctitis/proctosigmoiditis	2	2.82
Left-sided ulcerative colitis	2	2.82
Total studies	71	100

### 3.3 Effects on disease activity index

In evaluating the impact of probiotics, prebiotics, and synbiotics on patients with IBD, researchers rely on a suite of specialized disease activity indexes which are designed to provide a standardized measure of disease severity and response to treatment, encompassing a wide range of symptoms and clinical signs specific to IBD conditions. Since the introduction of the Disease Activity Index in 1955, many indexes have been developed over the years (36–39). A few commonly employed include the Crohn’s Disease Activity Index (CDAI), Harvey-Bradshaw Index (HBI), Simple Clinical Colitis Activity Index (SCCAI), and Ulcerative Colitis Activity Index (UCAI). In this section, we grouped studies investigating the effects of probiotics, prebiotics, and synbiotics on various IBD disease activity indices.

Among the 41 studies reporting probiotic interventions, 18 utilized disease activity indices as primary or secondary outcomes (Table 1). Of these, 12 reported significant changes in disease activity indices following intervention with probiotics compared to control; 11 in patients with UC, of which five reported changes using UCDAI, three reported using Mayo scores, two using SCCAI, and one using CAI. Only Fan et al. (40) reported improvement in both UCAI and CDAI after probiotic treatment with *B. longum, L. acidophilus*, and *E. faecalis* in combination with mesalazine. The utilized probiotic varies among studies reporting improvement in UCDAI: while Tamaki et al. utilized *B. longum*, Sood et al. and Tursi et al. utilized a *Lactobacillus* cocktail, whereas Huang et al. and Chen et al. utilized both *Lactobacillus* and *Bifidobacteria*. Among studies reporting improvements in Mayo scores after probiotics, interventions utilized comprise of *Lactobacillus (L) reuteri* enema by Oliva et al. (41), *L. salivarius, L. acidophilus*, and *B. bifidus* by Palumbo et al., and *B. longum, L. acidophilus*, and *E. faecalis* by Li et al. and Bamola et al. (42) was one of two studies that investigated the impact of the probiotic *Bacillus clausii* using the SCCAI metric which showed a significant decrease for the UC group, while Huynh et al. utilized a mixed probiotic of multiple *Lactobacilli* and *Bifidobacteria* that also was associated with significantly decreased SCCAI indices post intervention. Lastly, Kato et al. showed an improvement in CAI measures after intervention with *B. breve, B. bifidum*, and *L. acidophillus*.

Out of the 13 studies assessing prebiotics (Table 2), nine did not exhibit significant decreases in disease activity scores, as measured by indices such as CDAI, HBI, and the Rachmilewitz score. Specifically, four studies using fructo-oligosaccharides (FOS) or related compounds did not show notable improvement in CDAI or clinical remission rates for Crohn’s disease. However, two studies yielded positive outcomes: Valcheva et al. reported that inulin-type fructans significantly reduced the total Mayo score by ≥3 points in patients with active ulcerative colitis, while Ikegami et al. found a significant decrease in the Clinical Activity Index (CAI) in patients with mild-to-moderate ulcerative colitis using the prebiotic 1-ketose. Conversely, three other studies, including those using lactulose and *Pistacia lentiscus*, either failed to demonstrate a significant reduction in CAI or only showed secondary improvements in quality-of-life measures. These mixed findings highlight the need for further research to clarify the therapeutic potential of prebiotics in managing IBD disease activity.

Finally, only two studies evaluate the impact of symbiotic interventions using disease activity scores (Table 3). For instance, Steed et al. (43) reported a significant decrease in CDAI scores using *Bifidobacterium longum* and Synergy 1 on patients with CD compared to those administered a placebo. Similarly, Amiriani et al. (44) observed a decrease in SCCAI scores among UC patients treated with Lactocare synbiotic in managing the severity of UC. Currently, the limited literature available suggests that symbiotics may play a beneficial role in mitigating the severity of IBD, but further studies are required to confirm this trend.

Overall, our review reveals a diverse landscape of studies focused on evaluating the impact of probiotics and prebiotics on IBD conditions using various indexes, with a limited number of investigations into symbiotic treatments. Within this spectrum of research, our analysis highlights significant variability in the effectiveness of these interventions when assessed with disease activity indexes, resulting in a range of outcomes across different studies.

### 3.4 Effect on inflammatory markers

Inflammatory markers are widely used tools for diagnosing or monitoring disease activity in IBD. While there is no ideal inflammatory marker, numerous studies aim to identify reliable, noninvasive biomarkers for IBD. Presently, the most commonly utilized markers are C-reactive protein (CRP) and fecal calprotectin (Fcal), which are reliable indicators of inflammation in IBD42. Other markers include Fecal lactoferrin and erythrocyte sedimentation reactant (ESR). In this section, we specifically analyze studies investigating the effects of probiotics, prebiotics, and synbiotics on IBD using inflammatory markers as assessment tools.

Among probiotic interventions (Table 1), five studies out of 41 assessed inflammatory markers, including CRP, Fcal, and serum cytokines, as primary or secondary outcomes. Of these, two studies specifically investigated *Lactobacillus acidophilus* as part of multi-strain probiotics. Shadnoush et al. reported that probiotic yogurt containing *L. acidophilus* and *B. animalis* significantly reduced serum levels of CRP and TNF-α, indicating an anti-inflammatory effect. Similarly, Yılmaz et al. observed a decrease in both CRP and ESR in patients consuming *L. kefiri*, highlighting its potential to reduce systemic inflammation. Conversely, Bjarnason et al., using a combination of *L. rhamnosus*, *L. plantarum*, *L. acidophilus*, and *E. faecium*, found no significant change in CRP or ESR, though Fcal levels significantly decreased in UC patients. Additionally, Hegazy et al. demonstrated that a mix containing *L. delbrueckii* and *L. fermentum* significantly decreased Fcal levels when administered alongside standard therapy in UC patients, suggesting an enhancement of therapeutic effects. Finally, while D’Incà et al. administered *L. casei* with 5-ASA, they observed significant reductions in TNF-α and IL-1β but no changes in CRP, underscoring variability in how different probiotics affect inflammation markers. Collectively, these findings suggest that while certain probiotics show promise in reducing inflammatory markers, responses may vary depending on strain composition and patient factors.

Among the prebiotic interventions, 4 out of 13 clinical trials measured outcomes related to inflammatory markers, specifically CRP and fecal calprotectin (Fcal), with varying results (Table 2). Two trials utilized FOS or FOS-enriched inulin to assess these markers in active Crohn’s disease (CD) patients. Benjamin et al. found no significant difference in CRP or Fcal levels between the FOS and placebo groups after a 4-week intervention. Similarly, Hedin et al., using a combination of inulin and oligofructose in CD patients, observed no notable changes in Fcal levels between patients and healthy sibling controls. By contrast, two other studies investigated inflammatory markers in ulcerative colitis (UC) using different prebiotics. Papada et al., who administered *Pistacia lentiscus* (mastiha), reported no significant change in Fcal or lactoferrin within the intervention group, though the placebo group experienced an increase in these markers over time, indicating a possible protective effect from the prebiotic. Meanwhile, Casellas et al., who assessed oligofructose-enriched inulin in active UC, documented a significant early reduction in Fcal, suggesting an anti-inflammatory effect. These results demonstrate the variability in prebiotics’ efficacy across inflammatory markers and patient subgroups, emphasizing the need for further studies to clarify their role in IBD management.

Among synbiotic interventions (Table 3), two studies assessed their impact on inflammatory markers, yielding mixed results. Altun et al. investigated a synbiotic mix consisting of six probiotics (*Enterococcus faecium*, *Lactobacillus plantarum*, *Streptococcus thermophilus*, *Bifidobacterium lactis*, *Lactobacillus acidophilus*, and *Bifidobacterium longum*) alongside the prebiotic fructooligosaccharides (FOS), administered orally to patients with mild to moderate UC. This intervention significantly reduced CRP and ESR levels, indicating an anti-inflammatory effect. In contrast, Amiriani et al. examined a commercially available synbiotic mix, Lactocare, containing six probiotics (*Lactobacillus rhamnosus*, *Lactobacillus casei*, *Lactobacillus acidophilus*, *Bifidobacterium breve*, *Lactobacillus bulgaricus*, *Bifidobacterium longum*, and *Streptococcus thermophilus*) with FOS. This study reported no significant changes in inflammatory markers among subjects, suggesting variability in synbiotic efficacy. Overall, these mixed outcomes underscore the need for more targeted studies to determine the potential of synbiotics in reducing inflammation as a primary outcome in IBD management.

Overall, many studies have employed inflammatory markers as outcomes to investigate the impact of microbiome-based nutraceuticals. While a few combinations have demonstrated promise, there are inconsistent results within the same type of nutraceutical, with the majority of studies showing no significant change in inflammatory markers following intervention.

### 3.5 Effect on serum cytokines

Cytokines are crucial signaling proteins in the body that profoundly impact inflammation, playing a central role in the development and progression of IBD (45). These molecules are diverse, with functions ranging from promoting inflammation (proinflammatory cytokines) to reducing it (anti-inflammatory cytokines), and some can have dual roles depending on the context. This complex network includes extensively studied cytokines (i.e., TNF-α, INF-γ, IL-1, IL-4, IL-5, IL-6, IL-10, TGF-β) and more recently characterized cytokines (i.e., IL-12, IL-13, IL-18, IL-23) (46). The therapeutic management of IBD often includes pharmacological agents (e.g., anti-TNF-α agents) that target these cytokines directly, aiming to reduce their concentration and, consequently, the inflammatory response (47). Considering the role of cytokines as indicators of inflammation levels within the body, our focus here is on studies that have analyzed serum cytokine levels to gauge patient response to various treatments, including probiotics, prebiotics, and synbiotics.

Among probiotic interventions (Table 1), 9 out of 41 studies measured serum cytokines, including TNF-α, IL-6, IL-1β, and IL-10, to assess the anti-inflammatory effects of probiotics on IBD. Of these, 5 studies investigated TNF-α levels specifically: *Lactobacillus acidophilus* was part of a multi-strain intervention in two studies, both of which demonstrated a significant reduction in TNF-α. Studies using *L. casei* (two studies) also showed consistent decreases in TNF-α, while a single study on *Bacillus clausii* observed no significant change in this cytokine. Regarding IL-6, three studies evaluated its levels. In two studies using *B. clausii*, IL-6 levels were significantly reduced, indicating an anti-inflammatory effect; however, a single multi-strain study involving *L. acidophilus* found no significant change in IL-6 levels, indicating variability depending on probiotic composition. IL-1β levels were reported in four studies: *L. reuteri* and *L. casei* each appeared in one study and demonstrated significant reductions in IL-1β, whereas two studies using multi-strain formulations containing *L. acidophilus* yielded mixed results, with one showing a decrease in IL-1β and the other showing no effect. Finally, IL-10, an anti-inflammatory cytokine, was evaluated in three studies: two studies using *L. acidophilus* as part of multi-strain probiotics consistently showed increased IL-10 levels, while a single study using *B. clausii* did not report significant IL-10 modulation. In summary, mono-strain probiotics, particularly *L. casei* and *L. reuteri*, were consistent in reducing pro-inflammatory markers like TNF-α and IL-1β, whereas multi-strain probiotics with *L. acidophilus* demonstrated more varied outcomes. Additionally, studies involving *B. clausii* provided mixed results across cytokines, underscoring the influence of probiotic strain specificity on inflammatory responses in IBD patients.

Among the prebiotic interventions (Table 2), 4 out of 11 studies specifically measured serum cytokines, including IL-6 and IL-10, to assess anti-inflammatory effects on IBD. Of these, two studies examined IL-6 levels: Benjamin et al. utilized fructo-oligosaccharides (FOS) and observed a reduction in IL-6-positive lamina propria dendritic cells, indicating a positive anti-inflammatory response. However, Hedin et al., using inulin + oligofructose, reported no significant change in IL-6 levels, suggesting variability depending on prebiotic composition. Papada et al. and Casellas et al. reported effects on inflammatory markers but did not directly measure IL-6 levels. For IL-10, Benjamin et al. found an increase in IL-10, aligning with its anti-inflammatory role, while other studies did not assess IL-10 changes specifically. This systematic assessment indicates that fructo-oligosaccharides, particularly FOS, were associated with positive cytokine modulation (IL-6 reduction, IL-10 increase), while mixed results were seen with other prebiotics, such as inulin-based interventions. Overall, FOS demonstrated a more consistent anti-inflammatory effect across cytokines compared to inulin or multi-prebiotic interventions.

Among synbiotic interventions (Table 3), 4 out of 9 studies assessed serum cytokine changes to investigate anti-inflammatory effects in IBD. Steed et al. conducted a randomized, double-blind trial using a synbiotic containing *Bifidobacterium longum* with inulin/oligofructose, where biopsies from inflamed gut regions revealed high baseline TNF-α and IL-18 levels. After 3 months, the intervention group showed a significant reduction in TNF-α, while IL-18 levels remained elevated in non-inflamed tissue. Andrews et al. examined a combination of *Lactobacillus rhamnosus* and FOS, reporting a consistent decrease in IL-6 levels across inflamed regions. Similarly, Patel et al. observed a marked reduction in IL-1β in subjects receiving a multi-strain synbiotic including *Lactobacillus acidophilus*. By contrast, Smith et al. found no significant changes in IFN-γ or IL-10 levels with a bifidogenic synbiotic. In summary, interventions involving *B. longum* and multi-strain synbiotics demonstrated reductions in TNF-α and IL-1β, highlighting potential anti-inflammatory benefits, though the cytokine response varied by probiotic strain and combination used.

Overall, studies investigating the effect of probiotics, prebiotics, and synbiotics on cytokines as outcomes in patients with IBD show promise, highlighting significant reductions in inflammatory markers and anti-inflammatory cytokines.

### 3.6 Effect on microbiome composition

Given the strong relationship between the gut microbial composition and IBD pathogenesis, in this section we focus on studies that have investigated the influence of probiotics, prebiotics and synbiotics on the microbiome composition in patients with IBD.

In the context of probiotic interventions (Table 1), among the 20 studies assessing microbiome composition with probiotic interventions, *Lactobacillus* and *Bifidobacterium* species were used in 14 studies. Of these, eight studies reported increased abundance of both genera, with Bamola et al. and Shadnoush et al. showing significant increases in *Lactobacillus, Bifidobacterium*, and *Faecalibacterium* after *B. clausii* and multi-strain probiotic yogurt, respectively. Five studies using *Bifidobacterium breve* reported significant increases in *Bifidobacterium* species, with Kato et al. documenting specific increases in *B. breve* and *B. pseudocatenulatum*. However, three studies, including Ng et al., observed no significant shifts in overall bacterial abundance despite improvements in immune markers. Across studies, *Lactobacillus* and *Bifidobacterium* were consistently associated with positive shifts in microbiome composition, although clinical correlation varied. In a series of three placebo-controlled studies examining the administration of *Bifidobacterium breve, Bifidobacterium bifidum, Lactobacillus acidophilus* as probiotics, significant changes in intestinal flora before and after treatment were reported. First, Kato et al. (48) documented a significant increase in the fecal numbers of *Bifidobacterium breve* and *Bifidobacterium pseudocatenulatum* in the intervention group, which was not mirrored in the placebo group by the end of the trial (49). Matsuoka et al. (50) compared intestinal bacteria between groups experiencing relapse and those maintaining remission. They reported a significant decrease in *Bifidobacterium* species before relapse, suggesting a potential association between the concentration of *Bifidobacterium* species and disease activity (51). Another investigation found no differences between the probiotic and placebo groups in total fecal bacterial, *Bacteroidaceae*, or *Bifidobacteria* counts. However, after probiotic supplementation, there was a significant reduction in the relative proportion of *B. vulgatus* among all *Bacteroidaceae* (52). Additional clinical trials that utilized yogurt as a probiotic for patients with IBD revealed that the abundance of *Lactobacillus, Bifidobacterium*, and *Bacteroides* was significantly higher in the treatment group compared to the controls after the intervention (53, 54). Overall, the use of probiotics appears to influence the composition of the microbiome and, in some cases, normalize aspects of mucosal function to match those of healthy individuals, which may have implications for disease modulation and management.

Among prebiotic interventions (Table 2), 6 studies out of 11 focused on *Bifidobacterium longum* and *Ruminococcus* species. Three studies, including De Preter et al. and Joossens et al., using oligofructose-inulin combinations, reported increased *Bifidobacterium longum* and decreased *R. gnavus*, correlating with improved disease activity. Ikegami et al. found a reduction in alpha diversity with decreased *R. gnavus* in UC patients using 1-kestose compared to placebo. In contrast, two studies with inulin alone reported increased *Bifidobacterium* and *Lachnospiraceae* without clinical improvements. These findings suggest that while oligofructose-inulin prebiotics increase beneficial bacteria, effects on disease activity remain inconsistent.

Among the nine studies on synbiotic interventions that assessed microbiome composition (Table 3), five focused specifically on *Bifidobacterium* levels, with 3 of these studies reporting significant increases. Wilson et al. investigated the effects of a synbiotic containing *Bifidobacterium* and GOS in UC patients, finding significant increases in *Bacteroidaceae* but no significant changes in *Bifidobacterium* counts. In contrast, Steed et al. observed a notable increase in both *Bifidobacterium* and *Enterococci* with a similar synbiotic, highlighting differences in microbial outcomes with comparable formulations. Three studies, including a trial on children with CD receiving *Lactobacillus GG* and inulin, reported no significant shifts in *Lactobacillus* or overall microbial diversity between intervention and control groups. Across studies, synbiotics containing *Bifidobacterium* combined with various prebiotics, such as GOS and inulin, generally showed favorable increases in *Bifidobacterium* and *Enterococci* populations, though outcomes varied by formulation and patient group, underscoring the need for further exploration into strain-specific effects.

Overall, these studies reveal that probiotics have been the most extensively studied microbiome-targeting interventions for IBD. Most of these probiotic formulations are based on strains of *Bifidobacterium* and *Lactobacillus*. Prebiotics, in contrast, commonly consist of inulin and various oligosaccharides. Synbiotics, which combine the elements of probiotics and prebiotics, utilize a mix of *Bifidobacterium, Lactobacillus* strains, inulin, and oligosaccharides. Despite the lack of a consistent pattern in alterations of microbial composition following these interventions, there have been several instances where studies have demonstrated a positive link between microbial changes and improvements in disease activity among patients undergoing such treatments. This suggests that there is potential merit in continuing to investigate the effects of these interventions on microbiome compositions, holding promise for future therapeutic advancements in the management of IBD.

### 3.7 Effect on remission rate

Among studies assessing remission rates in IBD, 18 out of 30 probiotic interventions (Table 1) focused on UC, with the majority (12 studies) utilizing multi-strain mixtures. Of these, nine studies reported significant remission improvements with multi-strain probiotics. For example, Miele et al. found that 92.8% of pediatric UC patients receiving a high-dose multi-strain probiotic mixture, in addition to standard therapy, achieved remission compared to 36.4% in the placebo group. Similarly, Sood et al. evaluated a multi-strain mixture over 12 weeks in mild to moderate UC, with remission achieved in 42.9% of the intervention group versus 15.7% of controls, measured using the UCDAI. Across studies, combinations involving *Lactobacillus acidophilus*, *Bifidobacterium breve*, and *Escherichia coli* Nissle 1917 were associated with increased remission rates, supporting these strains’ potential in UC management. However, not all studies yielded significant effects on remission. Four trials, including Kruis et al. and Wildt et al., reported no improvement in remission rates in UC or CD, regardless of probiotic strain composition. These studies highlight the variability in remission outcomes, suggesting that strain selection and disease type are critical factors influencing probiotic efficacy in IBD.

Among prebiotic interventions (Table 2), only 1 out of 13 studies on remission reported significant outcomes. Ikegami et al. found that 1-kestose, a fructo-oligosaccharide variant, significantly improved remission rates in mild to moderate UC, with a 30% or greater decrease in Mayo scores in the treatment group, while no other prebiotic interventions reported similar remission outcomes. These findings suggest a limited but potentially specific role for certain prebiotics, such as FOS variants, in promoting remission in UC patients, though further study is needed.

In summary, multi-strain probiotic mixtures, especially those including *Bifidobacterium* and *Lactobacillus* species, show potential for inducing remission in UC, while prebiotics demonstrate less consistent efficacy. This variability emphasizes the importance of strain-specific formulations and targeted patient populations for optimizing remission outcomes in IBD.

### 3.8 Effect on relapse rate

In examining studies on relapse rates following probiotic and synbiotic treatments, we found 16 out of 30 probiotic studies (Table 1) that assessed relapse rates in IBD, particularly UC. Among these, nine studies reported significant decreases in relapse rates, especially with multi-strain probiotics and certain single-strain formulations. Yoshimatsu et al. evaluated a probiotic intervention in UC patients in remission, finding a 0% relapse rate in the probiotic group compared to 17.4% in the placebo group at 3 months, with the probiotic group continuing to show a lower relapse rate at 6 and 9 months, though not statistically significant. Similarly, Guslandi et al. reported a significant reduction in CD relapse with *Saccharomyces boulardii*, where 6.25% of the probiotic group relapsed compared to 37.5% in the control group after 6 months. Studies using *Escherichia coli* Nissle 1917 found comparable efficacy to mesalamine in preventing UC relapse, making it a viable option for patients unable to tolerate mesalamine. Despite these positive findings, seven studies, including those by Kruis et al. and Wildt et al., observed no significant effect of probiotics on relapse prevention in UC or CD, highlighting variability in efficacy likely due to differences in strain types, dosing, and patient characteristics. This inconsistency suggests that while certain probiotics may benefit IBD relapse prevention, effectiveness is not uniform across all formulations.

Regarding synbiotic interventions (Table 3), only 1 out of 7 studies investigated relapse rates, with Milek et al. reporting no significant difference in relapse between a group receiving inulin plus *Lactobacillus rhamnosus* GG and a control group. This limited evidence points to a need for further research on synbiotics to assess their potential role in IBD relapse prevention.

In summary, while probiotics, particularly multi-strain formulations and specific strains like *S. boulardii* and *E. coli* Nissle 1917, show promise in reducing relapse rates in UC and CD, there is less evidence supporting prebiotics or synbiotics in this area. Further research is necessary to clarify the conditions and formulations under which these interventions are effective in maintaining remission.

### 3.9 Side effect profile

Evaluating the safety profile and potential side effects of nutraceuticals such as probiotics, prebiotics, and synbiotics is crucial for determining the overall tolerability and risks associated with such treatments in managing gastrointestinal disorders such as IBD. The collected data from these studies indicate that while no severe adverse events or mortality have been linked to nutraceutical supplementation, there have been instances of minor side effects. For instance, within a trial involving the multi strain probiotic formulation, a small percentage of participants (12%) reported experiencing dizziness, flu-like syndromes, and abdominal bloating and discomfort (55). Similar observations of minor side effects were made in other research studies, suggesting a pattern that warrants consideration in medical decision-making with the patient but does not generally contraindicate the use of these nutraceuticals (48, 56–59). In summary, the absence of major adverse effects reinforces the potential of these interventions as relatively safe options for individuals seeking adjunctive therapies for gastrointestinal health, albeit with an acknowledgment of the possibility of minor discomforts that should be weighed on an individual basis.

### 3.10 Fecal microbiota transplantation

Lastly, we incorporate data from 11 clinical trials investigating the application of FMT in managing IBD, with most patients diagnosed with UC (Table 4). Most fecal transplants were delivered via enemas, while the most reported outcome measures included clinical response and remission rates. Moayyedi et al. (60) conducted a randomized clinical trial to determine the efficacy and safety of FMT delivered through rectal enemas in a cohort of 70 patients with active UC. The trial’s outcomes revealed that 24% of the patients treated with FMT achieved complete remission. This rate was significantly higher than the 5% remission observed in the placebo group, with no significant difference of serious adverse events compared to the placebo group. However, the authors identified several potential confounding factors within the study. Specifically, patients who were receiving immunosuppressive therapy and those who had more recently been diagnosed with UC exhibited higher remission rates. Additionally, most successful cases in the FMT group were associated with fecal material from a single donor, suggesting that FMT efficacy largely depends on the donor’s characteristics, explaining variable results across different cases. In a similar study, Rossen et al. (61) investigated the effects of FMT in 50 patients with mild to moderately active UC. Of these, 23 patients in the treatment group received FMT from healthy donors via a nasoduodenal tube, while 25 patients in the placebo group were administered autologous fecal microbiota. The response to the treatment was evaluated at the week 12 mark, revealing no significant differences in clinical or endoscopic remission between the treatment and placebo groups. Notably, serious adverse events associated with FMT were rare and not directly linked to the procedure. Interestingly, for those who reached remission, there was a notable shift in microbial profiles toward those of the healthy donors, and remission was associated with specific microbial compositions. However, the study utilized 15 different donors, making determining a super donor effect challenging due to the low number of procedures per donor (61). In a recent multicenter randomized open-label clinical trial, Březina et al. (62) evaluated the therapeutic efficacy of FMT in 45 patients diagnosed with active left-sided UC. Participants were randomized to receive either FMT or 5-aminosalicylic acid (5-ASA) enemas, with a follow-up period extending to 12 weeks. They reported that 57% of the patients in the FMT group achieved clinical remission by the end of week 12. In contrast, only 36% of those treated with 5-ASA reached remission. These results demonstrate that FMT is at least as effective as standard treatments, with a similar adverse effect profile. Notably, this study also demonstrated that FMT contributed to an increased diversity of the microbiome in recipients, suggesting that restoring microbial variety could play a crucial role in alleviating symptoms of left-sided UC (62). Furthermore, in a double-blind study, Paramsothy et al. (63), aimed to identify bacterial and taxonomic and metabolites in response to FMT. Eighty-one patients with active UC were randomly assigned to groups that received an initial colonoscopic infusion and then intensive multidonor FMT or placebo enemas, 5 d/wk. for 8 weeks. Patients in the FMT group received homogenized stoold from 3 to 7 unrelated donors. In this study, remission was defined as endoscopic remission (steroid free remission using mayo sub-score of 0). Patients with endoscopic remission presented with altered microbial diversity and composition. Furthermore, patients in remission after FMT had enrichment of *Eubacterium hallii* and *Roseburia inulivorans* compared with patients who did not achieve remission after FMT and had increased levels of short-chain fatty acid biosynthesis and secondary bile acids. Further into the effects of FMT on gut and fecal microbiota, a multi-center, double blind, placebo controlled was conducted to investigate the efficacy of FMT in total of 85 patients with active UC. The primary outcome was steroid-free clinical remission with endoscopic remission or response (Mayo score ≤ 2, all subscores ≤ 1, and ≥1-point reduction in endoscopy subscore) at week 8. The primary outcome was achieved in 27% of patients allocated to FMT versus 8% who were assigned to placebo. Microbiota analysis using 16 rRNA stool analysis. The analysis was done on fecal samples from 70 patients out of 85 total patients. Operational taxonomic units and phylogenetic diversity were significantly higher in the intervention group. Microbial diversity increased and persisted following FMT. Certain bacterial taxa were linked to clinical outcomes; notably, the presence of *Fusobacterium species* was associated with the absence of remission.

The cumulative findings from these trials suggest that FMT holds great promise as a treatment for UC, proving to be at least non-inferior to standard therapy with similar adverse effects. However, the complexity of microbial interactions and the variability among donors and patient response warrant further research, underscoring the necessity to fully understand and refine FMT as a therapeutic strategy before its routine clinical application.

## 4 Discussion

In recent years, research into the gut microbiome has illuminated its profound influence on human health and disease. Specifically, therapeutic modulation of the gut microbiome has emerged as a focal point of contemporary research in IBD. This surge in research activity has predominantly centered on nutraceutical interventions, such as probiotics, prebiotics, synbiotics, and FMT, owing to their non-inferior efficacy and favorable safety profiles compared to established pharmacological therapies. In this systematic review, we meticulously screened available literature, carefully filtering relevant studies investigating the effects of these interventions on patients with IBD and categorizing them according to one or more of eight reported outcome measures. Encompassing a total of 84 studies, our analysis constitutes a significant contribution to the body of knowledge on gut microbiome interventions in IBD.

The exploration of microbial-based therapeutic modalities has underscored the pivotal role of gut microbiota manipulation in managing IBD. Among these therapeutic strategies, probiotics and prebiotics are the most promising options, offering significant insights into gut health and disease management (64, 65).

Probiotics, in particular, have garnered attention for their potential to significantly disrupt the progression of IBD at various stages, making them a compelling study area. Researchers have pinpointed a range of promising strains, including *Enterococcus faecium, Bifidobacterium, Bacillus, S. boulardii*, various *Lactobacillus* strains, and *Pediococcus*, which appear to disrupt the disease’s progression at different stages (7, 66). These probiotics are thought to primarily exert their effects by outcompeting pathogenic bacteria for resources, thereby hindering their growth (67). Beyond this mechanism, probiotics are lauded for their ability to fortify the gut barrier, modulate inflammation, and bolster the host’s defense systems, all collectively supporting intestinal health (68). A crucial aspect of this beneficial impact is the production of short-chain fatty acid (SCFA) metabolites by healthy gut microbiota, including acetate, propionate, and butyrate (reference needed). These SCFAs have been shown to diminish the levels of pro-inflammatory agents such as lipopolysaccharides (LPS) and trimethylamine N-oxide (TMAO), which are implicated in the pathogenesis of IBD (69). Furthermore, probiotics have been observed to influence bile acid metabolism, potentially correcting imbalances associated with IBD (5, 70). Specifically, an alteration in the bile acid pool, characterized by increased levels of primary bile acids and decreased levels of secondary bile acids, has been noted in IBD patients and linked to the disease’s pathology (71, 72). The dysbiosis observed in IBD patients, characterized by a reduction in SCFA-producing bacteria, particularly from the *Ruminococcaceae* and *Lachnospiraceae* families within the *Firmicutes* phylum, further underscores the potential role of probiotics (5, 70). Probiotic bacteria target bile metabolism by increasing the levels of secondary bile acids, which confer beneficial effects on the gut mucosa (73). By targeting bile metabolism and increasing the levels of secondary bile acids, probiotic bacteria may confer protective effects on the gut mucosa offering a promising avenue for therapeutic intervention in IBD (74). On the other hand, prebiotics serve as SCFA inducers through bacterial fermentation pathways achieving optimal SCFA production hinges on several conditions, such as low pH and an optimal gut microbiome composition, both facilitated by probiotics (75). The significance of SCFAs, particularly in IBD management, lies in their beneficial effects on the epithelial barrier and the innate immune system. In IBD cases, the intestinal barrier is compromised, marked by the downregulation of epithelial cadherin within tight junctions, leading to decreased mucus thickness, altered goblet cell function—including mucin 2 and resistin-like molecule β (RELMβ)—, and impaired Paneth cell activity (1). This comprehensive approach to IBD therapy, leveraging the symbiotic relationship between probiotics and prebiotics, underscores the intricate connection between gut microbiota and overall intestinal health.

Several studies have demonstrated that both probiotics and prebiotics can enhance mucus production, facilitate tissue healing, and bolster the formation and distribution of tight junctions within the gut epithelium. Improving intestinal barrier function plays a vital role in attenuating metabolic diseases by upregulating the expression of claudin 1, GLP1, IL-10, occludin 1, and ZO-1 (6, 70, 74, 75). This results in an overall decrease in intestinal permeability, strengthening barrier defenses and functionality. Moreover, probiotics and prebiotics can improve the immunological aspect by upregulating anti-inflammatory markers such as IL-10 and TGF-β, while reducing the production of proinflammatory cytokines such as TNF-α in the intestinal mucosa of IBD patients (76). These actions are mediated by SCFAs, predominantly butyrate, which downregulates histone deacetylase (HDAC) and nuclear factor-κB (NFκB), thereby influencing the behavior of neutrophils, monocytes, macrophages, and gene expression (75). Several studies have indicated a strong association between IBD pathogenesis, oxidative stress, and DNA damage, and the antioxidant properties of probiotics are aimed at tackling this aspect. Probiotic bacteria have been shown to protect DNA from oxidative damage and enhance the activity of antioxidant enzymes in both *in vitro* and *in vivo* studies (77). The cumulative evidence underscores the vital role of probiotics and prebiotics in enhancing gut health, specifically through bolstering intestinal barrier function, modulating the immune response toward anti-inflammation, and protecting against oxidative stress, offering a promising therapeutic strategy to intervene at various points in the IBD pathogenesis.

Synbiotics, which are combinations of prebiotics and probiotics, are designed to harness the synergistic effects to restore a healthy balance of gut flora in various pathological conditions (78). Historically, the development of synbiotics was partly a response to the challenges associated with the implantation and survival of probiotics in the colon. Synbiotics prolong the survival of probiotics, thus enhancing their activity and immunomodulating abilities (79). This method decreases systemic inflammation by boosting the number of bacteria that produce SCFAs and supplying substrates for fermentation (78). Yang et al. (73) demonstrated that synbiotics can amplify beneficial gut bacteria populations and reduce coliform bacteria’s presence. Synbiotics can also elevate levels of key digestive enzymes, including lactase, sucrase, lipase, and isomaltase, providing insight into their synergistic effects. It is essential to recognize that the efficacy of synbiotic formulations can vary; for instance, Bruno et al. (80) reported that β-fructofuranosidase enzyme in *Bifidobacterium adolescentis* G1 displayed a preference for fructooligomers over inulin, a specificity shared by *B. bifidum*. Conversely, *B. longum* and *B. animalis* have been observed to hydrolyze various types of FOS and xylooligosaccharides (XOS), including those derived from inulin (80). In a recent study, Wang et al. (81) assessed the effects of probiotics, prebiotics, and synbiotics in an IBD-induced mouse model. Among all three interventions, synbiotics proved most efficacious in enhancing occludin expression (a proxy for barrier function) and reducing IL-6 levels over the long term. Synbiotics also exhibited the most significant improvements in disease markers, occludin expression, and inhibition of phosphorylated STAT3 (p-STAT3) in a model of chemically induced IBD (82). Additionally, mice receiving synbiotic treatment exhibited elevated IL-10 levels compared to other treatment groups. This evidence suggests that combining probiotics and prebiotics in a synbiotic formulation may offer the most substantial therapeutic benefits by leveraging the advantages of both (82). However, further exploration in this area is required, as methodological and logistical challenges constrain current research.

This systematic review found that the most commonly utilized probiotic formulation was a proprietary blend of eight bacterial strains. These include four *Lactobacillus* species (*L. paracasei, L. plantarum, L. acidophilus*, and *L. delbrueckii* subspecies *bulgaricus*), three *Bifidobacteria* species (*B. longum, B. breve*, and *B. infantis*), and *S. thermophilus*. The literature highlights various ways this mixture can positively impact gut health. Notably, these eight strains work together in a distinct yet synergistic manner to uphold the integrity of the intestinal barrier. Specifically, *S. thermophilus* plays a key role in bolstering host defense mechanisms. In contrast, *Bifidobacteria* species contribute to enhancing barrier integrity, and *Lactobacilli* are crucial for the production of signaling proteins that are essential for maintaining gut homeostasis (83). A recent study highlighted that a surface protein in *L. acidophilus* was particularly effective in restoring intestinal immunity and homeostasis (84). In another review, Cheng et al. (84) posited that multi strain probiotic mentioned impacts four key facets of the intestinal barrier: the mechanical, biological, chemical, and immune components (85). Mechanically, the mixture boosts the function of tight junction proteins such as occludin and zonula occludens-1, while downregulating claudin-2 (86). This improves tight junction protein function through increased T-cell protein tyrosine phosphatase activity, mitigating T-cell protein tyrosine phosphatase-dependent interferon-γ signaling and augmenting transepithelial electrical resistance (86). Additionally, the probiotic mixture has been shown to activate the mitogen-activated protein kinase p42/44 and p38 pathway, further increasing transepithelial electrical resistance (87). From a biological perspective, the mixture elevates the levels of commensal bacteria and decreases fungal populations (88). Chemically, it is known to upregulate genes such as MUC2, MUC3, and MUC5AC, regulating mucus secretion (87). Immunologically, the mixture inhibits the proinflammatory nuclear factor-κB (NF-κB) pathway, stimulates heat shock protein (HSP) production, and diminishes monocyte chemoattractant protein-1 (MCP-1) via early proteasome inhibition. It also enhances the peroxisome proliferator-activated receptor α (PPARα) signaling pathway, thereby counteracting the NF-κB pathway (89). Furthermore, the multi strain mixture has been found to promote dendritic cell (DC) maturation, inhibits interferon-inducible protein-10 (IP-10) in intestinal epithelial cells, and suppress lipopolysaccharide (LPS)-induced chemokine expression by inhibiting STAT-1 phosphorylation (90). It reduces the production of IL-12 (p40) in response to LPS, stimulates IL-10 production by DCs, and reduces the influx of CD11b + innate immune cells and CD4+/CD8+ adaptive immune cell traffic (91). The additional downregulation of the Toll-like receptor (TLR) signaling pathway also contributes to strengthening the intestinal immune barrier (92). In summary, the multi-strain probiotic shows great promise as an adjunct in patients with IBD in clinical trials, outcomes that are supported by mechanical, biological, chemical, and immunological mechanisms of action.

Determining the optimal blend and dosage of probiotics for effectively addressing various health conditions remains elusive. Typically, the recommended administration of probiotics to patients falls within the range of 10^8^–10^9^ colony-forming units (CFU) daily. This dosage is posited to guarantee the survival of approximately 10^6^ viable cells during transit to the intestines, a quantity deemed sufficient to impact the host’s health positively (93). Evidence synthesized from the studies encompassed in this review aligns with the recommended administration, suggesting that daily consumption of 10^9–10^ CFU of probiotics is correlated with clinically relevant modifications in the disease activity index for individuals suffering from IBD. This is consistent with findings of similar reviews, which have recommended an average of ≥10^9^ CFU per g regarding remission induction, relapse, and complication rate reductions (94). Chen et al. explored the dose–response relationship of 10^6^ to 10^10^ CFU/day of conjugated linoleic acid (CLA)-producing *B. breve* CCFM683 in DSS-induced mice and reported that 10^10^ and 10^9^ CFU/day of CCFM683 improved colitis symptoms. These improvements were assessed by DAI scores, reduced colon shortening, and preservation of colonic tissue integrity. These effects were not seen with lower dosages (95). These benefits were comparable or superior to those achieved with mesalazine treatments, with 10^10^ and 10^9^ CFU/day of CCFM683 significantly mitigating colonic damage and preserving goblet cells and crypts. Overall, the authors recommend a gavage dose of at least 10^8.65^ CFU/day to relieve colitis in DSS-induced mice (95). In a double-blind clinical trial, *E. coli* Nissle enemas were administered to patients with moderate distal UC activity, revealing a dose-dependent efficacy of the probiotic. Enemas containing 40 mL *E. coli* Nissle were more effective than those with 10 and 20 mL (96). On the other hand, oligofructose-enriched inulin emerged as the prebiotic of choice, with daily 15-20 grams typically offering clinical benefit across various disease indices.

As detailed in our review, FMT has demonstrated effectiveness across various human and animal studies (97), successfully inducing clinical and endoscopic remission and response in a subset of patients with IBD. Similar to our findings, a recent Cochrane review investigating the effects of FMT on IBD concluded that “FMT may increase the proportion of people with active UC who achieve clinical and endoscopic remission”; however, the certainty of evidence regarding remission maintenance and other outcomes in CD was low (98). Compared to its use in patients with rCDI, for which it was recently approved by the US FDA, when utilized for IBD, more than one infusion and careful selection of donor feces is often required to maintain remission or maintain relapse-free disease state (98). Although the precise mechanism remains unclear, FMT appears to correct IBD-associated gut dysbiosis and reduce inflammation. A post-clinical trial analysis conducted by Paramsothy et al. (63, 99) revealed that patients in remission following FMT exhibited an increased enrichment of *Eubacterium hallii* and *Roseburia inulivorans*, in contrast to those who did not achieve remission. This finding underscores the significance of specific microbiota in disease pathogenesis. Additionally, while the diversity of the microbiome was observed to increase post-FMT, the presence of *Fusobacterium* subspecies was found to be associated with a failure to achieve remission, suggesting a potential link between specific species and clinical outcomes. The authors also identified specific bacteria and metabolic pathways linked to the initiation of remission, which could prove beneficial in formulating future therapies for UC that are based on targeted microbial components. Furthermore, no significant difference was reported regarding side effects when comparing controls to intervention groups, suggesting that donor fecal transplant does not differ in its safety profile compared to allogenic FMT (63).

The variation in the results of studies investigating FMT can largely be attributed to differences in the delivery methods, including the frequency of treatments, discrepancies in community microbial composition, and the density of introduced bacterial populations. A significant challenge in FMT research involves establishing standardized fecal donor screening and selection protocols. Ideally, such protocols would account for donor factors like age, gender, and health status to maximize compatibility and safety. Additionally, the field requires standardized FMT procedures, encompassing the preparation of fecal material, dosing strategies, treatment regimens, and evaluation metrics for post-transplantation outcomes. Financial limitations also affect the breadth of clinical trials, particularly restricting comprehensive pre-trial analyses of donor microbial profiles, often conducted via 16S rRNA sequencing. Such analyses are crucial as they provide a comparative baseline for the microbiota of donors and recipients. Understanding the microbial match between donors and recipients is essential. This insight is pivotal for predicting FMT success rates, streamlining the transplantation process, and ultimately increasing the therapeutic potential of the treatment (5).

Although microbiomes also consist of fungi, we have not included them in this study. Among those studied, *Hericium erinaceus* (HE), a traditional edible mushroom, is known as a medicine food homology to ameliorate gastrointestinal diseases (100). The extract of this fungus has shown promising results in IBD pathogenesis. Among the studies, Diling et al. (101) prepared HE extracts (polysaccharide, alcoholic extracts and whole extracts) to be administrated for 2 weeks in rats with IBD induced by trinitro-benzene-sulfonic acid (TNBS) enema (150 mg/kg). It was found that there was an improvement in damage activity, common morphous, and tissue damage index scores in the colonic mucosa, along with a reduction in MPO activity. In IBD rats, the expression of inflammatory factors in the colonic mucosa varied, with increased levels of serum cytokines, Foxp3, and interleukin (IL)-10, while NF-κB p65 and tumor necrosis factor (TNF)-α levels were decreased. Additionally, T cells were activated, particularly in the group treated with alcohol extracts (102). Furthermore, it was found that the composition of gut microbiota in the *H. erinaceus* extracts-treated groups changed significantly when compared with the model group. Further studies revealed that the polysaccharides in HE extracts may play a prebiotic role, whereas the alcoholic extracts show bactericidin-like and immunomodulatory effects. All in all, showing promising results. In another study, Gravina et al. investigated the anti-inflammatory potential of a nutraceutical compound of HBQ-Complex® (*H. erinaceus*, berberine, and quercetin), biotin, and niacin in inflammatory bowel disease patients. The results showed that HBQ-Complex® (with the addition of niacin and biotin) decreased the expression of proinflammatory cytokines at the mRNA and protein levels in IBD tissue. On the contrary, mRNA and protein expression of the anti-inflammatory cytokine IL-10 showed a progressive increase (103). Future studies should also focus on the role of such fungi to highlight a novel therapeutic intervention in the management of IBD.

## 5 Limitations

This study underscores various limitations, particularly the heterogeneous nature of patient demographics, disease stages, symptoms, previous treatments, and progression, which complicates the assessment and synthesis of the examined therapies’ effects. A significant obstacle is the need for a standardized approach to fecal microbiome analysis, which prevents directly measuring the impact on gut microbiota. This gap underlines the urgent requirement for further placebo-controlled, randomized studies with prolonged follow-ups to link gut microbiota to IBD’s development more accurately. Quality control in probiotic formulations emerges as another significant issue, highlighted by discrepancies between product labeling and actual contents, contamination risks, and the use of inferior bacterial strains. Moreover, the efficacy of a probiotic can vary, even within the same strain, due to batch-to-batch variations stemming from inconsistent bacterial culturing methods employed by different manufacturers.

While probiotics have undergone extensive study and their benefits are well-documented, the scientific community recognizes a significant knowledge gap regarding prebiotics, synbiotics, and their synergistic effect in bolstering probiotics. This gap suggests untapped potential for therapeutic interventions that could complement or enhance the efficacy of probiotics. Despite the promising outcomes of FMT in treating IBD, its widespread adoption is hindered by healthcare professionals’ limited familiarity with its use, effectiveness, and safety concerns. Enhancing healthcare providers’ knowledge about FMT could significantly impact patient outcomes, encouraging its broader acceptance and potential integration into standard treatment protocols. Nonetheless, while FMT has shown effectiveness in IBD treatment, ongoing safety concerns, particularly regarding its safety profile, highlight the need for better documentation and reporting of adverse events to ensure patient safety. This may include understanding the specific metabolic pathways of different bacterial strains and species, which may help elucidate how to target specific gut organisms for health benefits.

## 6 Future perspectives

**Figure 4:**
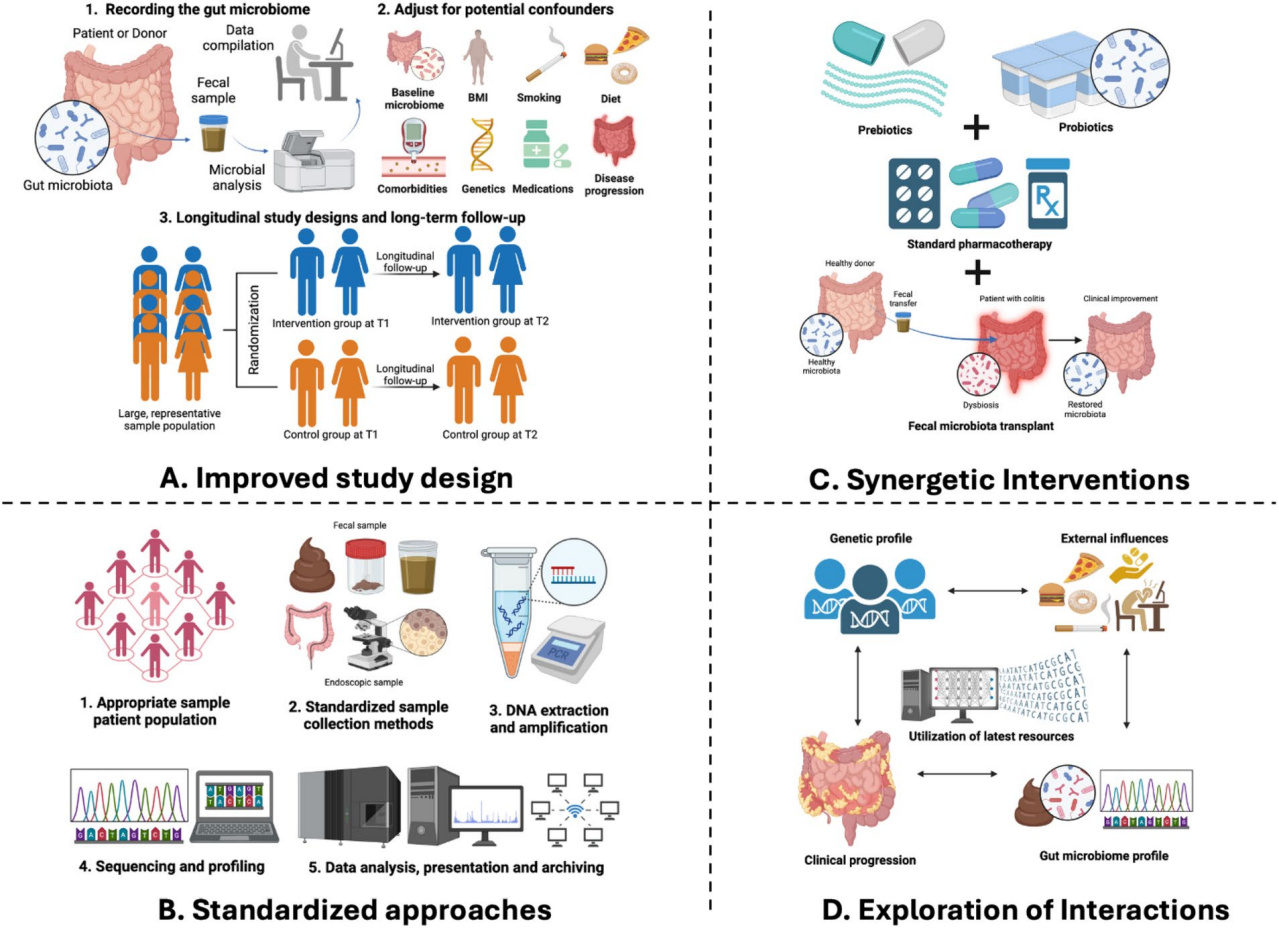
Future perspectives of gut microbiome research in IBD. **(A)** An improved study design will help addressing systematic biases and confounding variables by a thorough recording of fecal microbial composition, adjusting for potential confounders as well as implementing longitudinal study designs to explore the temporal relationships between microbiome shifts and the onset or progression. **(B)** Standardized approaches by adopting standardized approaches for inclusion and exclusion criteria, sample collection, processing, and data analysis which minimizes technical variability and facilitates meaningful cross-study comparisons. **(C)** Exploration of interactions between the gut microbiome and various factors—genetic, environmental, and clinical factors which holds immense promise for unraveling the pathophysiology of IBD. **(D)** Synergetic interactions by integrating microbiome modifications with existing preventive and therapeutic measures which presents a promising avenue for optimizing disease management in IBD.

This section addresses the previously mentioned limitations and proposes strategies to enhance future research in this field. Our discussion centers on potential improvements to study protocols and methodologies, emphasizing the need for standardized reporting across studies (Figure 4).

### 6.1 Improved study design

For IBD research involving probiotics, prebiotics, synbiotics, and FMT, we emphasize the importance of rigorous study design to effectively address systematic biases and confounding variables. This includes a thorough recording of fecal microbial composition and counts at both the beginning and end of trials, which is crucial for determining if clinical or endoscopic changes align with alterations in the gut microbiome, a key mechanism of action for these therapies. Additionally, in the context of FMT studies, documenting donors’ clinical and microbial profiles is vital to describe the therapy’s outcomes.

We also recommend implementing longitudinal study designs to explore the temporal relationships between microbiome shifts and the onset or progression of IBD. Such designs are instrumental in differentiating between correlation and causation and identifying predictive biomarkers that pave the way for targeted and personalized treatment plans.

Finally, we emphasize the importance of conducting studies with sufficient statistical power to overcome the limitations of small sample sizes, which can lead to challenges in microbiome research due to variability between different studies. To address this issue, we encourage the use of population-based cohorts and the inclusion of incident IBD cases in studies. Doing so can help ensure that the findings are robust, applicable to a broader population, and less prone to bias, thereby enhancing the potential for translating research findings into practical applications or treatments.

### 6.2 Standardized approaches

We recommend standardizing methodologies across all studies to improve the reliability and comparability of microbiome research results. Adopting standardized approaches for inclusion and exclusion criteria, sample collection, processing, and data analysis minimizes technical variability and facilitates meaningful cross-study comparisons. For instance, establishing standard protocols for sample collection kits, storage conditions, and sequencing methodologies can mitigate discrepancies arising from methodological heterogeneity. Moreover, leveraging ontological frameworks for metadata collection enhances data interoperability and facilitates meta-analyses, thereby maximizing the utility of microbiome data in IBD research.

### 6.3 Exploration of interactions

Unlocking the intricate interactions between the gut microbiome and various factors—genetic, environmental, and clinical—holds immense promise for unraveling the pathophysiology of IBD. By integrating multiomics data and harnessing the power of advanced analytical tools, including artificial intelligence, researchers are empowered to study the complex interplay between host genetics, environmental exposures, microbial dysbiosis, and disease phenotypes. Studying the interactions between genes, the microbiome, and the environment allows researchers to better understand how diseases develop and to find new potential treatments. However, because these interactions can be very complex and studies often have limited sample sizes, it may be difficult for researchers to account for all these factors properly when comparing different patient groups. In such cases, we recommend focusing on a subset of patients with similar characteristics across various factors, potentially yielding more reliable and informative results. Ultimately, we recommend categorizing patients with IBD based on their microbiome profiles and clinical characteristics. This may allow healthcare providers to develop personalized treatment strategies that are tailored to each individual’s unique disease course and response to treatment.

### 6.4 Synergistic interventions

Integrating microbiome modifications with existing preventive and therapeutic measures presents a promising avenue for optimizing disease management in IBD. By harnessing the modifiability of the gut microbiome, researchers and clinicians can explore innovative strategies to augment conventional treatments and improve patient outcomes. For example, interventions such as FMT or microbial-derived metabolites may complement existing pharmacological therapies by restoring microbial homeostasis and enhancing treatment efficacy. Additionally, synergistic approaches combining microbiome modulation with dietary interventions, other forms of biotics, or immunomodulatory agents hold the potential for personalized therapeutic regimens tailored to individual patient needs.

In conclusion, embracing meticulous study design, standardized methodologies, exploration of interactions, and synergistic interventions, are a few prospects that we believe would improve the quality of future research of microbiome therapy in IBD, as it holds immense promise for revolutionizing disease management and improving patient outcomes. Through interdisciplinary collaboration and innovative research endeavors, microbiome-based therapies have the potential to usher in a new era of precision medicine in the treatment of IBD.

## 7 Conclusion

This comprehensive systematic review evaluates 71 studies to discern the impact of probiotics, prebiotics, synbiotics, and FMT in patients with IBD. In a detailed examination of varied outcome measures—including the disease activity index, inflammatory markers, serum cytokines, microbiome composition, as well as the incidence of adverse effects, remission, and relapse rates—our review unfolds the complex therapeutic impact these treatments have through the modulation of the gut microbiota. The findings highlight the promising potential of therapeutic strategies that target gut dysbiosis as a pivotal aspect of treatment. While the results of this review are promising, it is crucial to note that further research is needed to fully understand the underlying mechanisms to overcome the limitations identified in this review. Such insights are imperative before these therapeutic strategies can be seamlessly incorporated into routine clinical practice for IBD management.

## Data Availability

The original contributions presented in the study are included in the article/Supplementary material, further inquiries can be directed to the corresponding author.

## Glossary

CAI: clinical activity index
CD: Crohn’s disease
CDAI: Crohn’s disease activity index
CFU: colony forming units
CRP: C-reactive protein
ESR: erythrocyte sedimentation rate
Fcal: fecal calprotectin
FMT: fecal microbiota transplant
FOS: fructooligosaccharides
GOS: galactooligosaccharides
HBI: Harvey-Bradshaw index
IBD: inflammatory bowel disease
MC: multicenter
MMDAI: Modified Mayo disease activity index
N/A: not applicable
N/R: not reported
RCT: randomized clinical trials
RDBPCT: randomized double-blind placebo controlled clinical trial
SCCAI: simple clinical colitis activity index
SCFAs: short chain fatty acids
TNF-α: tumor necrosis factor-alpha
UC: ulcerative colitis
UDCAI: Ulcerative disease activity index
