# Supplementary material for: Targeting gut microbiota dysbiosis in inflammatory bowel disease: a systematic review of current evidence
Source: Front Med (Lausanne). 2025 Feb 18;12:1435030. doi: 10.3389/fmed.2025.1435030 (PMC11876558; doi:10.3389/fmed.2025.1435030)
Supplement: Supplementary file 1 [file Data_Sheet_1.docx]

***Supplementary Appendix***

**Detailed search strategy**

**PubMed – Searched in August 2023**

("Inflammatory Bowel Diseases"[Mesh] OR "Inflammatory Bowel Diseas*"[tiab] OR “IBD” [tiab] OR "Crohn Disease"[Mesh] OR "Crohn Disease"[tiab] OR "Colitis, Ulcerative"[Mesh] OR "ulcerative colitis*"[tiab]) AND

(microbiome[tiab] OR "Gastrointestinal Microbiome"[tiab:~3] OR "gastrointestinal flora"[tiab:~3] OR "Gastrointestinal Microbiome"[tiab:~3] OR "microbes"[tiab] OR "gut microbes"[tiab:~3] OR "Microbiota"[tiab]) AND

("Nutraceuticals"[tiab] OR "prebiotic*"[tiab] OR "prebiotic"[tiab] OR "pre-biotic*"[tiab] OR "probiotic*"[tiab] OR "pro-biotic*"[tiab] OR "post-biotic*"[tiab] OR "synbiotic*"[tiab] OR "symbiotic*"[tiab] OR “Prebiotics” [MeSH Terms] OR "probiotics"[MeSH Terms] OR "synbiotics"[MeSH Terms] OR "short-chain fatty acid"[tiab] OR "short-chain fatty acids"[tiab] OR SCFA[tiab] OR SCFAs[tiab] OR acetate[tiab] OR butyrate[tiab] OR lactate[tiab] OR propionate[tiab] OR "microbial agent*"[tiab] OR "gut metabolites"[tiab] OR "bacterial metabolites"[tiab] OR postbiotic*[tiab] OR "non-viable probiotic*"[tiab] OR parabiotic*[tiab] OR paraprobiotic*[tiab] OR parapsychobiotic*[tiab] OR "ghost probiotic*"[tiab] OR metabiotic*[tiab] OR "microbial fraction*"[tiab] OR "functional protein*"[tiab] OR polysaccharide*[tiab] OR "teichoic acid"[tiab] OR muropeptide*[tiab] OR pili*[tiab] OR "heat-killed probiotic*"[tiab] OR "tyndallized probiotic*"[tiab] OR "bacterial lysate*"[tiab] OR lysate*[tiab] OR Streptococcus[tiab] OR Thermophilus[tiab] OR Bifidobacterium[tiab] OR Bacillus[tiab] OR Mycobacterium[tiab] OR Lactobacillus[tiab] OR Limosilactobacillus[tiab] OR Akkermansia[tiab] OR Lacticaseibacillus[tiab] OR Faecalibacterium[tiab] OR Bacteroides[tiab] OR Haemophiles[tiab] OR Streptococcus[tiab] OR Roseburia[tiab] OR Staphlycoccus[tiab] OR Moraxella[tiab] OR Arthrospira[tiab] OR Klebsiella[tiab] OR Eubacterium[tiab] OR Clostridium[tiab] OR Apilactobacillus[tiab] OR Saccharomyces[tiab] OR Escherichia[tiab] OR CLA[tiab] OR "conjugated linoleic acid"[tiab] OR PUFA[tiab] OR "polyunsaturated fatty acid"[tiab] OR FOS[tiab] OR Fructooligosaccharides[tiab] OR GOS[tiab] OR galactooligosaccharides[tiab] OR MOS[tiab] OR mannanoligosaccharide[tiab] OR XOS[tiab] OR Xylooligosaccharide[tiab] OR "Human milk oligosaccharides"[tiab] OR phenol*[tiab] OR "phenolic acid"[tiab] OR phytochemical*[tiab] OR polydextrose[tiab] OR inulin[tiab] OR "resistant dextrose"[tiab:~3] OR "α-linolenic acid"[tiab] OR "eicosapentaenoic acid"[tiab] OR "docosahexaenoic acid"[tiab] OR "linoleic acid"[tiab] OR "arachidonic acid"[tiab] OR "gamma-linolenic acid"[tiab] OR "resistant wheat starch"[tiab:~3] OR "resistant maltodextrin"[tiab] OR "resistant wheat"[tiab] OR "resistant starch"[tiab] OR "acacia gum"[tiab] OR "Acacia Senegal"[tiab] OR "Acacia fiber"[tiab] OR "soluble corn fiber"[tiab]) AND

("Diet"[Mesh] OR "Diet, Food, and Nutrition"[Mesh] OR Diet*[tiab] OR Nutrition*[tiab] OR Food[tiab] or Eating[tiab] OR Feed*[tiab] OR "Weight Loss"[tiab])

**Embase via OVID (1974 – 2023) Searched in August 2023**

1 | ((Inflammatory Bowel Diseas* OR IBD OR Crohn Disease OR ulcerative colitis*).ti,ab.) OR (exp Inflammatory Bowel Diseases/ OR exp Crohn Disease/ OR exp Colitis, Ulcerative/))

2 | (microbiome OR (Gastrointestinal ADJ3 Microbiome) OR (gastrointestinal ADJ3 flora) OR (Gastrointestinal ADJ3 Microbiome) OR microbes OR (gut ADJ3 microbes) OR Microbiota).ti,ab.

3 | ((Nutraceuticals OR prebiotic* OR prebiotic OR pre-biotic* OR probiotic* OR pro-biotic* OR post-biotic* OR synbiotic* OR symbiotic* OR short-chain fatty acid OR short-chain fatty acids OR SCFA OR SCFAs OR acetate OR butyrate OR lactate OR propionate OR microbial agent* OR gut metabolites OR bacterial metabolites OR postbiotic* OR non-viable probiotic* OR parabiotic* OR paraprobiotic* OR parapsychobiotic* OR ghost probiotic* OR metabiotic* OR microbial fraction* OR functional protein* OR polysaccharide* OR teichoic acid OR muropeptide* OR pili* OR heat-killed probiotic* OR tyndallized probiotic* OR bacterial lysate* OR lysate* OR Streptococcus OR Thermophilus OR Bifidobacterium OR Bacillus OR Mycobacterium OR Lactobacillus OR Limosilactobacillus OR Akkermansia OR Lacticaseibacillus OR Faecalibacterium OR Bacteroides OR Haemophiles OR Streptococcus OR Roseburia OR Staphlycoccus OR Moraxella OR Arthrospira OR Klebsiella OR Eubacterium OR Clostridium OR Apilactobacillus OR Saccharomyces OR Escherichia OR CLA OR conjugated linoleic acid OR PUFA OR polyunsaturated fatty acid OR FOS OR Fructooligosaccharides OR GOS OR galactooligosaccharides OR MOS OR mannanoligosaccharide OR XOS OR Xylooligosaccharide OR Human milk oligosaccharides OR phenol* OR phenolic acid OR phytochemical* OR polydextrose OR inulin OR (resistant ADJ3 dextrose) OR alpha-linolenic acid OR eicosapentaenoic acid OR docosahexaenoic acid OR linoleic acid OR arachidonic acid OR gamma-linolenic acid OR (resistant ADJ3 (wheat starch)) OR resistant maltodextrin OR resistant wheat OR resistant starch OR acacia gum OR Acacia Senegal OR Acacia fiber OR soluble corn fiber).ti,ab.) OR (exp Prebiotics/ OR exp probiotics/ OR exp synbiotics/)

4 | ((Diet* OR Nutrition* OR Food or Eating OR Feed* OR Weight Loss).ti,ab.) OR (exp Diet/ OR exp Diet, Food, and Nutrition/))

5 | #1 AND #2 AND #3 AND #4

**Cochrane Library – Searched in August 2023**

1 | (Inflammatory Bowel Diseas* OR IBD OR Crohn Disease OR ulcerative colitis*):ti,ab,kw

2 | MeSH descriptor: [Inflammatory Bowel Diseases] explode all trees

3 | MeSH descriptor: [Crohn Disease] explode all trees

4 | MeSH descriptor: [Colitis, Ulcerative] explode all trees

5 | (microbiome OR (Gastrointestinal near/3 Microbiome) OR (gastrointestinal near/3 flora) OR (Gastrointestinal near/3 Microbiome) OR microbes OR (gut near/3 microbes) OR Microbiota):ti,ab,kw

6 | (Nutraceuticals OR prebiotic* OR prebiotic OR pre-biotic* OR probiotic* OR pro-biotic* OR post-biotic* OR synbiotic* OR symbiotic* OR short-chain fatty acid OR short-chain fatty acids OR SCFA OR SCFAs OR acetate OR butyrate OR lactate OR propionate OR microbial agent* OR gut metabolites OR bacterial metabolites OR postbiotic* OR non-viable probiotic* OR parabiotic* OR paraprobiotic* OR parapsychobiotic* OR ghost probiotic* OR metabiotic* OR microbial fraction* OR functional protein* OR polysaccharide* OR teichoic acid OR muropeptide* OR pili* OR heat-killed probiotic* OR tyndallized probiotic* OR bacterial lysate* OR lysate* OR Streptococcus OR Thermophilus OR Bifidobacterium OR Bacillus OR Mycobacterium OR Lactobacillus OR Limosilactobacillus OR Akkermansia OR Lacticaseibacillus OR Faecalibacterium OR Bacteroides OR Haemophiles OR Streptococcus OR Roseburia OR Staphlycoccus OR Moraxella OR Arthrospira OR Klebsiella OR Eubacterium OR Clostridium OR Apilactobacillus OR Saccharomyces OR Escherichia OR CLA OR conjugated linoleic acid OR PUFA OR polyunsaturated fatty acid OR FOS OR Fructooligosaccharides OR GOS OR galactooligosaccharides OR MOS OR mannanoligosaccharide OR XOS OR Xylooligosaccharide OR Human milk oligosaccharides OR phenol* OR phenolic acid OR phytochemical* OR polydextrose OR inulin OR (resistant near/3 dextrose) OR alpha-linolenic acid OR eicosapentaenoic acid OR docosahexaenoic acid OR linoleic acid OR arachidonic acid OR gamma-linolenic acid OR (resistant near/3 (wheat starch)) OR resistant maltodextrin OR resistant wheat OR resistant starch OR acacia gum OR Acacia Senegal OR Acacia fiber OR soluble corn fiber):ti,ab,kw

7 | MeSH descriptor: [Prebiotics] explode all trees

8 | MeSH descriptor: [Probiotics] explode all trees

9 | MeSH descriptor: [Synbiotics] explode all trees

10 | (Diet* OR Nutrition* OR Food or Eating OR Feed* OR Weight Loss):ti,ab,kw

11 | MeSH descriptor: [Diet] explode all trees

12 | MeSH descriptor: [Diet, Food, and Nutrition] explode all trees

13 | #1 OR #2 OR #3 OR #4

14 | #6 OR #7 OR #8 OR #9

15 | #10 OR #11 OR #12

16 | #13 AND #5 AND #14 AND #15

**Web of Science – Search in August 2023**

1 | (TS = ("Inflammatory Bowel Diseas*" OR IBD OR "Crohn Disease" OR "ulcerative colitis*") OR AB = ("Inflammatory Bowel Diseas*" OR IBD OR "Crohn Disease" OR "ulcerative colitis*"))

2 | (TS = (microbiome OR (Gastrointestinal NEAR/3 Microbiome) OR (gastrointestinal NEAR/3 flora) OR (Gastrointestinal NEAR/3 Microbiome) OR microbes OR (gut NEAR/3 microbes) OR Microbiota) OR AB = (microbiome OR (Gastrointestinal NEAR/3 Microbiome) OR (gastrointestinal NEAR/3 flora) OR (Gastrointestinal NEAR/3 Microbiome) OR microbes OR (gut NEAR/3 microbes) OR Microbiota))

3 | (TS = (Nutraceuticals OR prebiotic* OR prebiotic OR pre-biotic* OR probiotic* OR pro-biotic* OR post-biotic* OR synbiotic* OR symbiotic* OR "short-chain fatty acid" OR "short-chain fatty acids" OR SCFA OR SCFAs OR acetate OR butyrate OR lactate OR propionate OR "microbial agent*" OR "gut metabolites" OR "bacterial metabolites" OR postbiotic* OR "non-viable probiotic*" OR parabiotic* OR paraprobiotic* OR parapsychobiotic* OR "ghost probiotic*" OR metabiotic* OR "microbial fraction*" OR "functional protein*" OR polysaccharide* OR teichoic acid OR muropeptide* OR pili* OR "heat-killed probiotic*" OR "tyndallized probiotic*" OR "bacterial lysate*" OR lysate* OR Streptococcus OR Thermophilus OR Bifidobacterium OR Bacillus OR Mycobacterium OR Lactobacillus OR Limosilactobacillus OR Akkermansia OR Lacticaseibacillus OR Faecalibacterium OR Bacteroides OR Haemophiles OR Streptococcus OR Roseburia OR Staphlycoccus OR Moraxella OR Arthrospira OR Klebsiella OR Eubacterium OR Clostridium OR Apilactobacillus OR Saccharomyces OR Escherichia OR CLA OR "conjugated linoleic acid" OR PUFA OR "polyunsaturated fatty acid" OR FOS OR Fructooligosaccharides OR GOS OR galactooligosaccharides OR MOS OR mannanoligosaccharide OR XOS OR Xylooligosaccharide OR "Human milk oligosaccharides" OR phenol* OR phenolic acid OR phytochemical* OR polydextrose OR inulin OR (resistant NEAR/3 dextrose) OR "alpha-linolenic acid" OR eicosapentaenoic acid OR "docosahexaenoic acid" OR "linoleic acid" OR "arachidonic acid" OR "gamma-linolenic acid" OR (resistant NEAR/3 ("wheat starch")) OR resistant maltodextrin OR "resistant wheat" OR "resistant starch" OR "acacia gum" OR "Acacia Senegal" OR "Acacia fiber" OR "soluble corn fiber") OR AB = (Nutraceuticals OR prebiotic* OR prebiotic OR pre-biotic* OR probiotic* OR pro-biotic* OR post-biotic* OR synbiotic* OR symbiotic* OR "short-chain fatty acid" OR "short-chain fatty acids" OR SCFA OR SCFAs OR acetate OR butyrate OR lactate OR propionate OR "microbial agent*" OR "gut metabolites" OR "bacterial metabolites" OR postbiotic* OR "non-viable probiotic*" OR parabiotic* OR paraprobiotic* OR parapsychobiotic* OR "ghost probiotic*" OR metabiotic* OR "microbial fraction*" OR "functional protein*" OR polysaccharide* OR teichoic acid OR muropeptide* OR pili* OR "heat-killed probiotic*" OR "tyndallized probiotic*" OR "bacterial lysate*" OR lysate* OR Streptococcus OR Thermophilus OR Bifidobacterium OR Bacillus OR Mycobacterium OR Lactobacillus OR Limosilactobacillus OR Akkermansia OR Lacticaseibacillus OR Faecalibacterium OR Bacteroides OR Haemophiles OR Streptococcus OR Roseburia OR Staphlycoccus OR Moraxella OR Arthrospira OR Klebsiella OR Eubacterium OR Clostridium OR Apilactobacillus OR Saccharomyces OR Escherichia OR CLA OR "conjugated linoleic acid" OR PUFA OR "polyunsaturated fatty acid" OR FOS OR Fructooligosaccharides OR GOS OR galactooligosaccharides OR MOS OR mannanoligosaccharide OR XOS OR Xylooligosaccharide OR "Human milk oligosaccharides" OR phenol* OR phenolic acid OR phytochemical* OR polydextrose OR inulin OR (resistant NEAR/3 dextrose) OR "alpha-linolenic acid" OR eicosapentaenoic acid OR "docosahexaenoic acid" OR "linoleic acid" OR "arachidonic acid" OR "gamma-linolenic acid" OR (resistant NEAR/3 ("wheat starch")) OR resistant maltodextrin OR "resistant wheat" OR "resistant starch" OR "acacia gum" OR "Acacia Senegal" OR "Acacia fiber" OR "soluble corn fiber"))

4 | (TS = (Diet* OR Nutrition* OR Food OR Eating OR Feed* OR "Weight Loss") OR AB = (Diet* OR Nutrition* OR Food OR Eating OR Feed* OR "Weight Loss"))

5 | #1 AND #2 AND #3 AND #4

**Scopus – Search in August 2023**

( ( TITLE-ABS ( diet* OR nutrition* OR food OR eating OR feed* OR "Weight Loss" ) ) OR ( INDEXTERMS ( diet OR food OR nutrition ) ) ) AND ( ( TITLE-ABS ( nutraceuticals OR prebiotic* OR prebiotic OR pre-biotic* OR probiotic* OR pro-biotic* OR post-biotic* OR synbiotic* OR symbiotic* OR "short-chain fatty acid" OR "short-chain fatty acids" OR scfa OR scfas OR acetate OR butyrate OR lactate OR propionate OR "microbial agent*" OR "gut metabolites" OR "bacterial metabolites" OR postbiotic* OR "non-viable probiotic*" OR parabiotic* OR paraprobiotic* OR parapsychobiotic* OR "ghost probiotic*" OR metabiotic* OR "microbial fraction*" OR "functional protein*" OR polysaccharide* OR teichoic AND acid OR muropeptide* OR pili* OR "heat-killed probiotic*" OR "tyndallized probiotic*" OR "bacterial lysate*" OR lysate* OR streptococcus OR thermophilus OR bifidobacterium OR bacillus OR mycobacterium OR lactobacillus OR limosilactobacillus OR akkermansia OR lacticaseibacillus OR faecalibacterium OR bacteroides OR haemophiles OR streptococcus OR roseburia OR staphlycoccus OR moraxella OR arthrospira OR klebsiella OR eubacterium OR clostridium OR apilactobacillus OR saccharomyces OR escherichia OR cla OR "conjugated linoleic acid" OR pufa OR "polyunsaturated fatty acid" OR fos OR fructooligosaccharides OR gos OR galactooligosaccharides OR mos OR mannanoligosaccharide OR xos OR xylooligosaccharide OR "Human milk oligosaccharides" OR phenol* OR phenolic AND acid OR phytochemical* OR polydextrose OR inulin OR ( resistant W/3 dextrose ) OR "alpha-linolenic acid" OR eicosapentaenoic AND acid OR "docosahexaenoic acid" OR "linoleic acid" OR "arachidonic acid" OR "gamma-linolenic acid" OR ( resistant W/3 ( "wheat starch" ) ) OR resistant AND maltodextrin OR "resistant wheat" OR "resistant starch" OR "acacia gum" OR "Acacia Senegal" OR "Acacia fiber" OR "soluble corn fiber" ) ) OR ( INDEXTERMS ( prebiotics OR probiotics OR synbiotics ) ) ) AND ( ( TITLE-ABS ( "Inflammatory Bowel Diseas*" OR ibd OR "Crohn Disease" OR "ulcerative colitis*" ) ) OR ( INDEXTERMS ( "Inflammatory Bowel Diseases" OR "Crohn Disease" OR "Ulcerative Colitis" ) ) ) AND ( TITLE-ABS ( microbiome OR ( gastrointestinal W/3 microbiome ) OR ( gastrointestinal W/3 flora ) OR ( gastrointestinal W/3 microbiome ) OR microbes OR ( gut W/3 microbes ) OR microbiota ) )
